# Age-stratification reveals age-specific intestinal microbiota signatures in juvenile idiopathic arthritis

**DOI:** 10.1186/s40348-024-00186-6

**Published:** 2024-12-10

**Authors:** Lisa Budzinski, Toni Sempert, Leonie Lietz, René Maier, Gi-Ung Kang, Anne Sae Lim von Stuckrad, Carl Christoph Goetzke, Maria Roth, Aayushi Shah, Amro Abbas, Katrin Lehman, Kathleen Necke, Stefanie Bartsch, Ute Hoffmann, Mir-Farzin Mashreghi, Robert Biesen, Tilmann Kallinich, Hyun-Dong Chang

**Affiliations:** 1 German Rheumatology Research Center Berlin – A Leibniz Institute, Charitéplatz 1, Berlin, 10117 Germany; 2 Department for Cytometry, Institute of Biotechnology, Technische Universität Berlin, Berlin, Germany; 3 Department of Pediatric Respiratory Medicine, Immunology and Critical Care Medicine, Charité Campus Virchow, Charité Universitätsmedizin Berlin, Berlin, Germany; 4 German Center for Child and Adolescent Health (DZKJ), Partner Site Berlin, Berlin, Germany; 5 Department of Rheumatology and Clinical Immunology, Charité Campus Mitte, Charité Universitätsmedizin Berlin, Berlin, Germany

**Keywords:** JIA, Microbiome, Microbiota phenotyping, Flow cytometry, 16S rRNA sequencing, Age

## Abstract

**Objective:**

Juvenile Idiopathic Arthritis (JIA) comprises diverse chronic inflammatory conditions driven by malfunction of the immune system. The intestinal microbiota is considered a crucial environmental factor correlating with chronic inflammatory diseases, and for JIA certain alterations in the microbiota have already been described.

**Methods:**

Here, we have characterized intestinal microbiota samples from 54 JIA patients and 38 pediatric healthy controls by conventional 16S rRNA sequencing and by single-cell analysis for phenotypic features by multi-parameter microbiota flow cytometry (mMFC), which complements the population-based taxonomic profiling with the characterization of individual bacterial cells.

**Results:**

We found age to be a crucial confounder in microbiota analyses of JIA patients. Age stratification revealed specific microbiota alterations neglected by the general comparison of JIA patients and pediatric controls.

**Conclusion:**

Age groups presented distinct taxonomic profiles and microbiota phenotypic signatures which transitioned with age, highlighting changes in the microbiota-immune system interaction with age.

**Supplementary Information:**

The online version contains supplementary material available at 10.1186/s40348-024-00186-6.

## Introduction

Juvenile idiopathic arthritis (JIA) comprises the most common chronic inflammatory diseases in children and teenagers, which principally affect joints but show diverse additional manifestations and a gender bias towards females [1, 2]. Rapid diagnosis and treatment are crucial to prevent irreversible damage of joint cartilage and tissue, but also treated patients are at increased risk to suffer from long-lasting chronic inflammation resulting in joint and bone deformations or involving other organs [3, 4]. Despite the urgency, causes and the pathogenesis of the heterogeneous set of diseases of JIA remain poorly understood. An important environmental factor implicated in many chronic inflammatory diseases is the intestinal microbiome. Several studies have analyzed the intestinal microbiota of children with JIA [5–8].

However, despite the finding that age was often determined to be a major confounder in previous studies of the JIA-associated microbiome [7], the role of age in JIA-associated microbiota studies was so far not resolved. This is surprising as both the microbiota as well as the immune system of infants and children is considered to be subject to very dynamic changes [9, 10]. Here, we took a closer look at the correlation between age and the microbiome in JIA. We performed an age-stratified microbiome characterization in a JIA cohort and have identified significant age-related alterations in the microbiome between JIA patients and their corresponding age-matched controls, which were masked when comparing the cohorts in an unstratified manner. Further, we have extended the 16S rRNA-based analysis of the intestinal microbiome by single-cell phenotyping of the bacteria to interrogate features of the microbiome-host interaction as well as adaption processes of the bacteria [11–13]. Using flow cytometry, we assessed the microbial community structure and diversity with quantitative DNA staining and light scatter measurement. In addition, we analyzed the coating of bacterial cells with host immunoglobulins of different isotypes and measured the expression of specific surface sugars by the bacteria. We could show important shifts in host-microbiome interaction and identify several age-specific taxonomic alterations in JIA. Our data highlight the importance of patient stratification and the benefit of complementing taxonomic results with single-cell phenotyping.

## Materials and methods

### Subjects

We recruited 54 patients with JIA (Table 1) from the Department of Pediatric Respiratory Medicine, Immunology and Critical Care Medicine at the Charité – Universitätsmedizin Berlin between October 2020 and September 2023. Age- and sex-matched pediatric healthy controls (Table 1) were recruited during the same period. Patients with rheumatoid arthritis and adult healthy controls (Table 2) were recruited between February 2021 and April 2022 and served as controls. All participants or their legal guardians gave written informed consent prior to sample collection according to the approval of the local ethics committee of the Charité Berlin (EA2/113/20).

**Table 1:** Clinical characteristics of JIA and pediatric healthy controls including age groups

**Variable**	**Cohort**	**Total**	**Age group (yrs)**		
			**group 1 (1-5)**	**group 2 (6-11)**	**group 3 (>= 12)**
**Cohort size**	JIA	54	16	21	17
	pHC	38	13	16	9
**Mean age (yrs, ± SD)**	JIA	8.89 ± 4.78	3.25 ± 1.18	8.57 ± 1.83	14.59 ± 1.97
	pHC	7.92 ± 4.57	3.15 ± 1.21	8.13 ± 1.78	14.44 ± 1.88
**Sex**					
Female	JIA	34 (62.96)	11 (68.75)	12 (57.14)	11 (64.71)
	pHC	23 (60.53)	9 (69.23)	8 (50.00)	6 (66.67)
Male	JIA	20 (37.04)	5 (31.25)	9 (42.86)	6 (35.29)
	pHC	15 (39.47)	4 (30.77)	8 (50.00)	3 (33.33)
**JIA subtype**					
Oligoarthritis^a^	JIA	34	12	13	9
Polyarthritis^b^		12	4	5	3
Psoriasis arthritis^c^		3	0	1	2
Enthesitis-associated arthritis^d^		5	0	2	3
**cJADAS10 score (mean ± SD)**	JIA	8.68 ± 4.84	6.69 ± 3.67	9.43 ± 4.29	9.62 ± 6.02
**Mean disease duration (months ± SD)**	JIA	37.5 ± 43.6	12.9 ± 17.2	34.9 ± 36.4	64.0 ± 53.2
**Disease activity**					
inactive /mild (IDa/MiDa)	JIA	6	2	2	2
moderate (MoDA)		42	14	15	13
high (HDa)		6	0	4	2
**Treatment **(DMARDS & glucocorticoids)					
naive	JIA	6	1	4	1
glucocorticoid (local)		24	9	7	8
glucocorticoid (systemic)		1	1	0	0
antibiotics + glucocorticoid (local)		1	0	1	0
cDMARD		1	0	1	0
cDMARD + glucocorticoid (local)		8	2	4	2
cDMARD + glucocorticoid (systemic)		1	0	1	0
cDMARD + glucocorticoid (systemic + local)		4	2	0	2
bDMARD + glucocorticoid (local)		2	0	0	2
bDMARD + cDMARD		1	0	1	0
bDMARD + cDMARD + glucocorticoid (local)		5	1	2	2
**Antibiotic treatment**^**e**^					
treated	JIA	15	7	5	3
untreated		33	7	12	14
undocumented		6	2	4	0

^a^affects up to four joints, pre-dominantly knees, ankles or elbows^3^

^b^affects five or more joints^3^

^c^presents additional rashes of the skin^3^

^d^affects sites of attachment between bone and muscles, ligaments and tendons^3^

^e^Within the last two years before sample collection

**Table 2:** Demographic characteristics of each cohort (JIA, pediatric controls, RA and adult controls)

Cohort	Cohort size	mean age (yrs, ± SD)	Sex	
			**female (%)**	**male (%)**
**JIA**	54	8.89 ± 4.78	34 (62.96)	20 (37.04)
**Pediatric healthy controls**	38	7.92 ± 4.57	23 (60.53	25 (39.47)
**RA**	16	59.5 ± 9.4	13 (81.25)	3 (18.75)
**Adult healthy controls**	18	44 ± 12.39	11 (61.11)	7 (38.89)

### Collection and preparation of stool samples

All participants provided a stool sample of about 1-2g, collected in a stool sampling tube (Sterilin™, Thermo Fisher Scientific). The samples were immediately transferred to 4°C and kept at that temperature for a maximum of 96 h before further processing. When longer storage was required, samples were directly frozen at −80°C. The time between stool collection by the participants and transfer to 4°C ranged between 0 and 24 h. Stool samples were processed as described in [12]. In short, each sample was diluted in autoclaved and 0.2 µm sterile-filtered PBS (Steritop® Millipore Express®PLUS 0.22 µm, Cat. No: 2GPT05RE) to a concentration of 100 mg/ml. The suspension was sequentially filtered through 70 µm (Falcon, Cat. No. 352350) and 30 µm filters (CellTrics®, Sysmex). 10 µl of each sample was subsequently stored at −20°C for later 16S rRNA gene sequencing. The remaining sample was frozen at −80°C in 40% glycerol with a defined OD (0.4).

### Multi-parameter Microbiota Flow Cytometry (mMFC)

Frozen microbiota stocks of 0.4 OD were topped up with 1 mL of autoclaved and sterile-filtered PBS and centrifuged at 13,000 × g for 10 min, 4 °C. The pellet was incubated in 500 µl blocking solution containing 20 µg/ml mIgG1 (clone: IS5-21F5, Miltenyi Biotech Cat. No.: 130–106–545) and 10 µg/ml mIgG2a (clone: S43.10, Miltenyi Biotech Cat. No.: 130–106–546) in PBS for 5 min at RT. The suspension was topped with 1.5 ml PBS and subjected to another centrifugation step (13,000 × g, 10 min, 4 °C). The pellets were re-suspended in PBS containing 0.2% BSA (v/w) and 25 µg/µl DNase (Sigma Aldrich Cat. No. 10104159001), which was also used as staining buffer. Cell density was adjusted to 0.02- 0.04 OD_690_/ml. 100 µL of the cell suspension was used per staining panel, i.e. stained with the immunoglobulin or the lectin panel. For the immunoglobulin panel, the antibodies used were anti-human IgM-Brilliant Violet 650 (clone: MHM-88, Biolegend® Cat. No. 314526), anti-human IgG-PE/ Dazzle™ 594 (clone: HP6017, Biolegend® Cat. No. 409324), anti-human IgA1-Alexa Fluor 647 (clone: B3506B4, Southern Biotech Cat. No. 9130–31), anti-human IgA2-Alexa Fluor 488 (clone: A9604D2, Southern Biotech Cat. No. 9140–30). The lectins used for staining were 0.5 µg/test of Peanut Agglutinin-CF®488 (PNA, Biotium Cat. No.29060), 0.5 µg/test of Concanavalin A-CF®680 (Con A, Biotium Cat. No. 29020–1), 0.25 µg/test of Wheat Germ Agglutinin-CF®555 (WGA, Biotium Cat. No.29076–1) and 0.5 µg/test of biotinylated Solanum Tuberosum Agglutinin (STL, Vector Laboratories/Biozol Cat. No. B-1165) shortly pre-incubated with 2 µL (1:50, v/v) anti-Biotin-PerCP antibody (clone: Bio3-18E7, Miltenyi Biotech Cat. No. 130–133–293). The tests were incubated for 30 min at 4 °C and subsequently topped up with 1 ml of 5 µM Hoechst solution (Hoechst 33,342, Thermo Fisher Scientific Cat. No. 62249) and incubated for another 30 min at 4 °C. The samples were washed with 900 µl PBS/BSA and re-suspended in fresh PBS/BSA after centrifugation for acquisition. All samples were acquired on a *BD Influx™ cell sorter (Becton–Dickinson).* For each sample, 3 × 10^5^ Hoechst 33,342-positive events (mean fluorescence intensity > 10) were recorded. We controlled the staining procedure by including a standardized microbiota sample (anchor sample) comprising a pool of different donors.

### Clustering of flow cytometric data

Raw FCS-files from flow cytometry were imported to R without any transformation using FlowCore’s read.flowSet [14] and were gated in the environment of the flowWorkspace [15] to reduce instrument noise by including only events > MFI 1 for forward (FSC) and side scatter (SSC). A self-organizing map (SOM, kohonen package) [16, 17] was trained on a data set comprising 3 × 10^5^ cells, subsampled and concatenated from approx. 300 individuals to capture the expected diversity of mMFC patterns, stained for host immunoglobulin coating and surface sugar expression of the bacteria by the som() function of the kohonen package for a 5 × 5 hexagonal map segmenting the data based on gaussian neighborhood. For SOM training on the 3 × 10^5^ cells subsample clusters should contain at least 0.05% of the cells which yielded 2025 cluster for each staining panel. To characterize the phenotypic diversity of samples used in this study, we then mapped the pre-determined cluster to each sample by kohonen::map() after down sampling to 3 × 10^4^ cells per individual in favor of computing power and time, as we found 3 × 10^4^ cell to represent a sample sufficiently. Each sample is now described by an individual abundance of cells in each cluster. The combination of the two sets of clusters (immunoglobulin and lectin panel) resulted in the full description of a sample for its microbiota phenotype. Thus, in total each microbiota sample is represented by the distribution of 6 × 10^4^ cells among 4050 clusters for further analysis.

The data for the t-SNE projection was processed using the default settings of Rtsne package [18].

### Feature selection

Normalized mMFC cluster counts and relative abundances of bacterial genera, respectively, were the starting point to refine differences in cohort comparisons. Briefly, all data were pre-filtered to exclude non-significant features with *p* > 0.05, Wilcoxon rank-sum test (vegan package [19]), removing all mMFC clusters and 16S rRNA sequencing-derived taxonomic units not contributing to the discrimination between the cohorts. In a second filtering step, Recursive Feature Elimination (RFE) was applied to remove weak features for classification, with rfeControl() function in caret package [20]. The importance of the selected features was obtained within the RFE according to the consensus ranking through the tenfold cross-validation. The resulting features were then selected from the entire set of data (taxonomic profiles or phenotypic profiles) and all samples evaluated for that selection mainly by the diversity matrix of the Bray–Curtis dissimilarity which was then projected as distances by principal component analysis (see section: data evaluation and plotting).

### 16S rRNA sequencing (Illumina MiSeq platform)

For 16S rRNA gene sequencing, we amplified the V3/V4 region of the 16S rRNA gene (for: TCGTCGGCAGCGTCAGATGTGTATAAGAGACAGCCTACGGGnGGCWGCAG, rev: GTCTCGTGGGCTCGGAGATGTGTATAAGAGACAGGACTACHVGGGTATCTAATCC [21]; TIB MOLBIOL Syntheselabor GmbH) directly from a microbiome sample with a prolonged initial heating step of 5 min. After the amplicon PCR the genomic DNA was removed by AmPure XP Beads (Beckman Coulter Life Science Cat. No. A63881) with a 1:1.25 ratio of sample to beads (v/v). The amplicons were checked for their size and purity on a 1.5% agarose gel, and if suitable, subjected to the index PCR using the Nextera XT Index Kit v2 Set C (Illumina, FC-131–2003). After Index-PCR, the samples were cleaned again with AmPure XP Beads (Beckman Coulter Life Science Cat. No. A63881) in a 1:0.8 ratio of sample to beads (v/v). Samples were analyzed by capillary gel electrophoresis (Agilent Fragment Analyzer 5200) for correct size and purity with the NGS standard sensitivity fragment analysis kit (Agilent Cat. No. DF-473). Of all suitable samples a pool of 2 nM was generated and loaded to the Illumina MiSeq 2500 system.

### Sequence alignment Illumia MiSeq data & batch correction

Paired-end reads generated by Illumina MiSeq 16S rDNA sequencing were filtered and trimmed using Trimmomactic (Version 0.39) [22]. 7 leading bases with qualities below 35 were trimmed and reads shorter than 180 bases were filtered out. Using the DADA2 (Version 1.22.0) software package [23], forward and reverse reads were truncated at 260 and 210 bases respectively and filtered with a minimum quality score of 12 and a maximum of 0 ambiguous nucleotides. Amplicon sequence variants (ASVs) were identified using the default settings of the DADA2 algorithm and ASVs were classified using the Silva 138.1 prokaryotic SSU taxonomic training data formatted for DADA2 [24]. After alignment of the sequences with DECIPHER (Version 2.24.0) [25], a phylogenetic tree was computed using FastTree (Version 2.1.11) [26]. For analysis the ASVs were matched with the respective phylogenetic information, the data was processed to species level and normalized using the decostand(method = “total”) function from vegan (version 2.6.4). Species were filtered for a prevalence of 5% and low-abundant species (less than 0.01%) were excluded. The *MMUPHin::adjust_batch*function was applied for batch effect correction [27].

### Data evaluation and plotting

Statistical analyses were implemented through R (v. 4.0.3 or later versions), unless stated otherwise (suppl. Methods). Computation of β-diversity (Bray–Curtis dissimilarity) was computed using vegdist(data, method = ”bray”) function from vegan package [19]. The Bray–Curtis dissimilarity is a statistical metric to quantify the difference in composition between two cohorts. In our case it is the abundance of cells in each cluster or the presence or absence of bacterial taxa and their abundance. The Bray–Curtis dissimilarity is defined by a number between 0 and 1, 0 indicating that two samples/cohorts are completely similar and 1 indicating that two samples/cohorts do not share anything. The PCoA was computed by the R base function cmdscale() on the respective distance matrix followed by Adonis test adonis() from vegan package to evaluate the variance within groups. Graphical representation of the dissimilarity of all samples by Principal Coordinates Analysis (PCoA) was plotted using ggplot2 package. Statistical comparisons of paired data points by stat_compare_means() using ggpubr package. The abundance of bacterial taxa or cluster and their enrichment, represent by generalized fold change, were calculated by applying the SIAMCAT package [28]. The generalized fold change, a pseudo-fold change, is calculated as the mean value of the difference between the quantiles across two groups/conditions.

## Results

### JIA patients are differently well characterized by general taxonomic and phenotypic alterations in the intestinal microbiome

We have characterized the intestinal microbiome of 54 juvenile idiopathic arthritis (JIA) patients in comparison to 38 pediatric healthy controls (pHC) (Table 1) by conventional 16S rRNA sequencing and multi-parameter microbiota flow cytometry (mMFC, Supp. Figure 1). We determined the microbiota phenotype of an individual by clustering the flow cytometric data into 2025 clusters of phenotypically similar bacterial cells per panel (Supp. Figure 1A). The combination of both sets of clusters to 4050 clusters (2025 from the immunoglobulin panel and 2025 from the agglutinin panel) represented the phenotypic microbiota fingerprint of an individual. Based on the fingerprint, we determined the beta-diversity between samples using the Bray–Curtis dissimilarity index. This index ranges between 0 (samples are the same in composition and distribution) and 1 (samples are completely distinct in composition and distribution) and presents the congruence between samples projected as distances in a principal coordinate analysis plot (Supp. Figure 1B1). The further samples are away from each other, the more different these are in the taxonomic composition or the phenotypic features of their intestinal microbiota, respectively (Supp. Figure 1B4). We filtered the features, i.e. taxa or phenotypic clusters, for statistical significance between the two groups by the Wilcoxon test and selected only clusters with *p* < 0.05 (Supp. Figure 1B2). The statistically significant features were then subjected to recursive feature elimination to select the set of features describing the differences between the cohorts at best and most robustly (Supp. Figure 1B3).

**Fig. 1:**
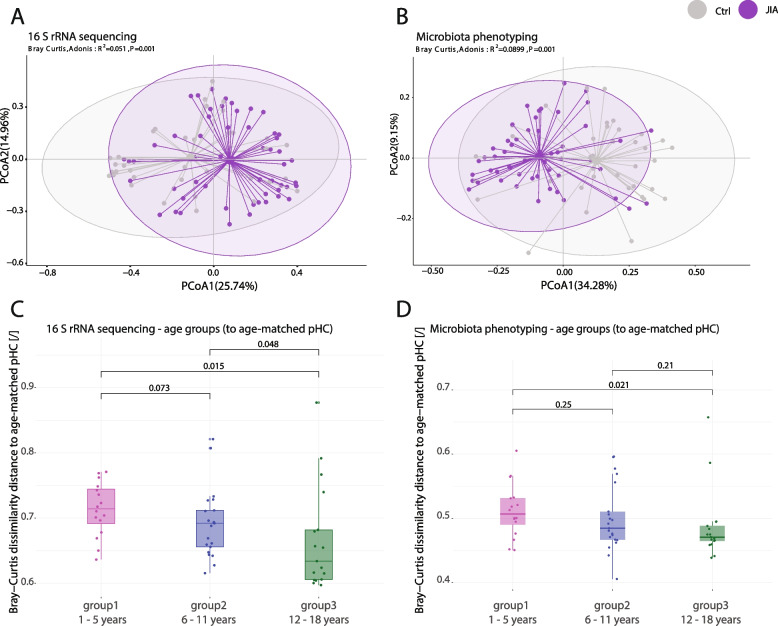
JIA presents taxonomic and phenotypic alterations compared to pediatric controls in the intestinal microbiome. (**A, B**) PCoA of the beta-diversity by Bray–Curtis index of all samples of the JIA cohort (*n* = 54 individuals, purple) and healthy controls (*n* = 38 individuals, grey) for the selected taxonomic signature (**A**, 28 features) or phenotypic signature (**B**, 166 clusters) of the intestinal microbiome for the comparison JIA vs. healthy pediatric controls. R^2^ and ANOVA test for each group. (**C, D**) Box plots for the distance of each patients Bray–Curtis beta-diversity index to the mean beta-diversity index of the age-matched healthy control group, evaluated for the JIA vs. HC intestinal microbiome signature in taxonomy **(C)** or microbiota phenotype **(D)**. Grouped for age groups: age group 1: 1 – 5 years, age group 2: 6–11 years, age group 3:12–18 years. Box plot show median, upper hinge: 75th percentile, lower hinge 25th percentile of data, Wilcoxon test, unpaired

Considering all features, JIA and pHC showed significant differences in taxonomic composition and the microbiota fingerprint (Supp. Figure 2A, B). The cohort separation between our JIA cohort and the pHCs was significantly improved, when we filtered the features as described (Supp. Figure 1B), identifying 28 taxa and 166 phenotypic clusters describing the differences between JIA and pHC at best (Fig. 1A, B; *p* < 0.001). 16 taxa were enriched in JIA e. g. *Parabacteroides sp*., *Blautia sp*. and *UBA1819 sp.,* while these patients showed lower abundances for different *Bacteroides sp.* and *Lachnospiraceae* (Supp. Figure 3). The JIA microbiota phenotype signature mainly comprised clusters with differences in surface sugar expression by the intestinal bacteria. 21 clusters out of the 166 clusters referred to immunoglobulin coating (Supp. Figure 4A). In detail, the clusters elevated in abundance for JIA contained bacteria with low to intermediate scatter and DNA signals, indicating smaller cells, and low coating with host immunoglobulins, primarily with IgA2 (Supp. Figure 4A, purple). Clusters derived from the agglutinin panel which are enriched in JIA patients showed a broad distribution in scatter signals and DNA staining, but had high STL and WGA staining, indicating elevated expression of N-acetyl-glucosamine, as well as PNA staining, marking galactose residues (Supp. Figure 4B, purple). Several clusters were enriched in healthy controls that referred to lectin labelling with higher signal intensities than clusters enriched in JIA (Supp. Figure 4B, grey). The low R^2^-value of the PCoA analyses, 0.051 for 16S rRNA sequencing and 0.097 for mMFC, based on the beta-dissimilarity of all samples for the 166 clusters or 28 taxa indicated that still the differences between JIA and healthy controls were not well captured by the selected features (Fig. 1A, B).

We controlled for potential confounders in this analysis and observed that both signatures were not confounded by disease severity as the different disease activity groups (inactive/ mild disease activity: IDa/MiDa, moderate disease activity (MoDa) and high disease activity: HDa) did not show significant difference in their distance to healthy controls (Supp. Figure 5A, B). We next analyzed whether age is a confounder and stratified the cohort into 3 age groups (age group 1: 1 – 5 years, age group 2: 6 – 11 years, age group 3: 12 – 18 years), before applying the taxonomic and phenotypic signatures to the respective groups. We observed that the signatures were still separating controls and patients of age group 1 (16 S rRNA sequencing: distance median > 0.7, microbiota phenotyping distance median > 0.5). However, the signatures did not well apply to age groups 2 and 3. For age groups 2 and 3, the differences between controls and patients became less clear (16 S rRNA sequencing median distances < 0.7, mMFC median distances < 0.5).

In summary, we could identify a signature generally differentiating our JIA cohort from pHCs. However, this signature did not apply to all patients similarly well.

### The microbiome in JIA shows distinct taxonomic patterns with age

**Fig. 2:**
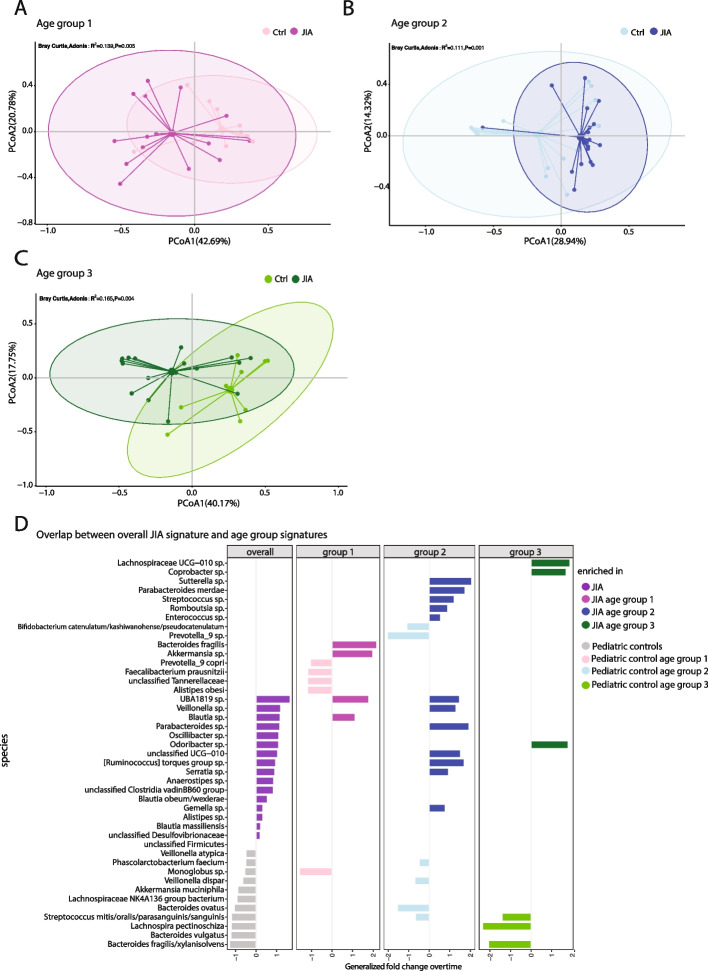
JIA presents distinct taxonomic alterations in the intestinal microbiome with age. (**A, B, C**) PCoA of the beta-diversity by Bray–Curtis index for age-matched comparisons of JIA patients and healthy controls: (**A**) Age group 1: 1 – 5 years, 16 patients (magenta), 13 healthy controls (pink), 9 taxa. (**B**) Age group 2: 6 – 11 years, 21 patients (dark blue), 16 healthy controls (light blue), 18 taxa.(**C**) Age group 3: 12 – 18 years, 17 patients (dark green), 9 healthy controls (light green), 6 taxa. R^2^ and ANOVA test for each group. (**D**) List of all taxa selected to describe JIA patients in comparison to healthy controls, either as one group or dissected for age. Generalized fold change > 0, enriched in JIA cohorts, generalized fold change < 0, enriched in control cohorts. Black lines indicate taxa that occur in more than one signature

We investigated the potential to improve the cohort separation and therefore the patient characterization by stratifying the patients and controls into the three age groups prior to identifying differentiating signatures the comparison of the taxonomic composition. For the definition of these groups we considered not only a similar distribution regarding sample size but also to the different life stages of children assumed to impact the microbiota and the immune system and correlating to previous finding of age as a confounder in microbiome studies in JIA [7]. We indeed found age-group specific taxonomic signatures between JIA and pHCs that allowed an improved distinction compared to the overall cohort comparison without age stratification (Fig. 2A-C, R^2^ = 0.111 – 0.165). We identified 9 taxa which were significantly different in JIA for children of 1 – 5 years old (Supp. Figure 6A). Two elevated taxa were found overlapping with the general JIA signature namely *Blautia sp.* and *UBA 1819 sp*. while five taxa elevated in a healthy pediatric microbiome overlapped (Fig. 2D). *Akkermansia sp*. and *Bacteroides fragilis* were uniquely enriched in age group 1 patients (Fig. 2D). The comparison of JIA patients and pHCs in age group 2 identified 18 taxa (Supp. Figure 6B), eleven of which were already identified in the general JIA signature. Seven of the overlapping species are elevated in JIA patients: *UBA 1819 sp*., *Parabacteroides sp., Veilonella sp., Ruminococcus torques group sp., unclass. UCG-10, Serratia sp*. and *Gemella sp*.. The species *Sutterella sp., Parabacteroides merdae, Streptococcus sp., Romboutsia sp.* and *Enteroccocus sp.* were exclusively enriched in JIA patients of age group 2*.* We only found a low number of taxa characteristic for age group 3 (3 taxa, Supp. Figure 6C) but which resulted in the best cohort separation (Fig. 2C, R^2^ = 0.165, PCoA1: 40.17%, PCoA2: 17.75%). *Lachnospiraceae UCG-010 sp*. and *Coprobacter sp.* were exclusively enriched in age group 3. The age groups showed no overlap among each other for the taxa enriched in JIA, but only for some taxa increased in pHCs (Fig. 2D). Of note, the selected taxa could be found in many individuals of the respective cohort, but never in all individuals (Supp. Figure 6A-C).

Our results highlight that age-associated microbiome signatures were not part of the JIA signature identified across the entire cohort. Instead, the JIA patients displayed unique, disease-related taxonomic alterations with age while the healthy children showed less change in their microbiome composition with age.

### The JIA microbiota phenotype is distinct in different age groups

**Fig. 3:**
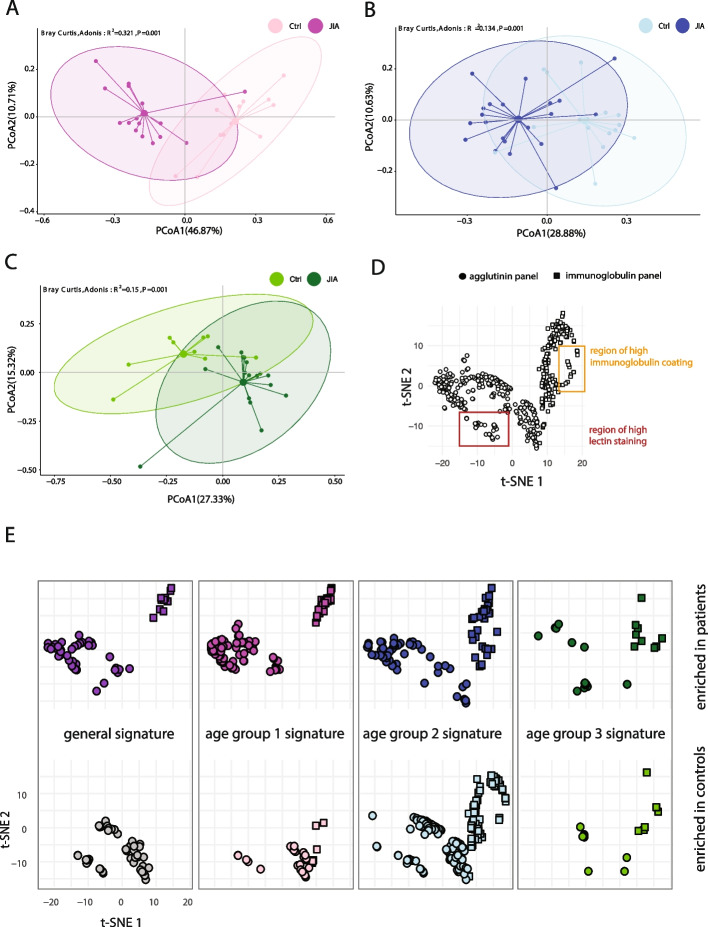
The intestinal microbiota phenotype in JIA is hallmarked by age-specific and by age transitioning cellular features of bacteria. (**A, B, C**) PCoA of the beta-diversity by Bray–Curtis index for age-matched comparisons of JIA patients and healthy controls: (**A**) Age group 1: 1 – 5 years, 16 patients (magenta), 13 healthy controls (pink), 139 phenotypic clusters. (**B**) Age group 2: 6 – 11 years, 21 patients (blue), 16 healthy controls (light blue), 244 phenotypic clusters. (**C**) Age group 3: 12 – 18 years, 17 patients (dark green), 9 healthy controls (light green), 34 phenotypic clusters. R.^2^ and ANOVA test for each group. (**D**) T-SNE computed for all the combination of selected phenotypic clusters from all comparisons: all JIA vs. all pediatric controls plus age-matched comparisons (524 clusters). Overview of all selected phenotypic clusters differentiated by panel (agglutinin panel: circle, immunoglobulin panel: quadrat) and definition of regions with high immunoglobulin coating or lectin staining. (**E**) Clusters of the overall comparison and each age-wise comparison highlighted. Clusters enriched in JIA are depicted in purple (overall signature), magenta (age group 1), blue (age group 2), dark green (age group 3), while and clusters enriched in controls are depicted in grey (overall signature), pink (age group 1), light blue (age group 2) and light green (age group 3)

We next tried to identify age group specific microbiota phenotypic signatures (Fig. 3A-C). We identified age group-specific features which greatly improved the discrimination between JIA patients from their healthy controls in the respective age group (age group 1: R^2^ = 0.321, 139 clusters, age group 2: R^2^ = 0.134, 244 clusters; age group 3: R^2^ = 0.15, 41 clusters; marker distribution in Supp. Figure 7). To compare the age group-specific signatures between each other and to the general JIA signature, we computed a t-SNE projection of all relevant clusters of all comparisons (Fig. 3D) and dissected the projection into clusters enriched in patients or controls for each age group (Fig. 3 E) and general expression for each marker (Supp. Figure 8). The signatures of the entire JIA cohort and that of age group 1 occupy similar regions in the t-SNE plot, comprising primarily lectin clusters (107 out of 139 clusters). Age group 1 and 2 share a number of immunoglobulin clusters. The age group 2 signature comprises the most clusters with the highest proportion of immunoglobulin clusters compared to all other groups (105 out of 244 clusters). Age group 3 is defined by a rather distinct group and a low number of clusters with little overlap to any other group. These t-SNE projections highlight the fact that the general JIA signature did not represent all patients equally well and neglected several phenotypic features of the age group specific, distinctive microbiota signatures.

### With age microbiome features of JIA transition towards RA-associated features

As we found age group 3 to be quite different to age group 1 and 2 in our analyses, we addressed the question, whether this age group would align more with adult rheumatoid arthritis (RA) patients than with JIA. To compare the JIA signatures with RA, we defined the taxonomic and phenotypic signatures discriminating RA patients (16 patients) from adult healthy controls (aHC, 18 individuals) (Supp. Figure 9 A, B). We identified 20 taxa best differentiating RA, five of which showed increased abundances in our RA cohort, namely *Colidextribacter sp.*, *Alistipes shahii, Oscillibacter sp., unclass. Clostridum methylpentosum group* and *Bacteroides sp*. (Supp. Figure 9 C). By microbiota phenotyping, 72 clusters were identified, which clearly distinguished RA patients from healthy controls (suppl. Figure 9B). Many of the clusters enriched in RA were characterized by high signals for IgA2 and IgM as well as STL and WGA (Supp. Figure 9D).

**Fig. 4:**
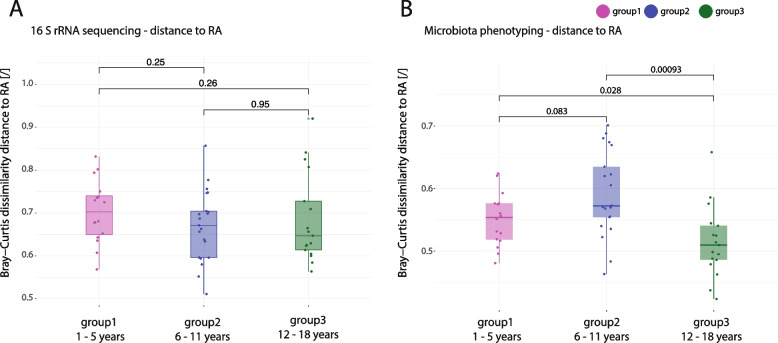
Microbiota features of RA become more evident in JIA patients with age. (**A, B**) Box plots for the distance in beta-diversity of each JIA patients, grouped by age, to the mean beta-diversity index of the RA cohort evaluated for (**A**) the taxonomic signature of RA and (**B**) the phenotypic signature of RA. Age group 1 (1- 5 years, magenta), age group 2 (6–11 years, blue), age group 3 (12–18 years, dark green). Wilcoxon test, unpaired

We next applied the taxonomic or phenotypic signature of RA to the JIA cohort and determined the distances in beta-diversity for each JIA patient to the mean beta-diversity of the RA cohort and grouped the results for the respective age groups (Fig. 4A, B). By taxonomic composition the JIA patients showed a mean Bray–Curtis dissimilarity distance of 0.71 in age group 1, 0.68 in age group2 and 0.65 in age group 3 to the RA patients according to the RA microbiome signature (Fig. 4A). The Bray–Curtis dissimilarity distance was not significantly different in the different JIA age groups. When using the phenotypic signature of RA, we observed that overall the Bray–Curtis dissimilarity distance was lower (Fig. 4B): 0.56 in age group 1, 0.57 in age group 2 and 0.51 in age group 3. In addition, JIA patients of age group 3 showed a significantly reduced distance to RA patients in taxonomy and phenotype compared to the other age groups, indicating that indeed the patients of age group 3 are more similar to RA regarding the microbiota phenotypic signature.

## Discussion

In this study we characterized the microbiome of 54 JIA patients and 38 pediatric healthy controls by conventional 16S rRNA amplicon sequencing and multi-parametric microbiota flow cytometry. We observed significant differences for the taxonomic as well as the phenotypic signature between patients and controls (Fig. 1B, C).

This study identified age as an important, non-negligible factor in intestinal microbiome studies of children. This should not be surprising as the intestinal microbiota is still very dynamic in childhood [9, 29–31]. From birth, it is estimated to take up to four years until an intestinal community stabilizes initially [9, 29, 30, 32]. In this initial phase microbiota-impacted immune system development occurs and disturbances of the microbiome in that phase have been associated with disease, including JIA [10, 33, 34].

In general, early colonization of the intestinal tract is dominated by Enterobacteriacea, Bifidobacteriaceae and Bacteriodaceae driven by e. g. breast feeding and individual environmental exposure [35, 36]. This composition shifts with age towards dominant proportions of bacteria coming from the phyla Bacillota and Bacteroidota which are maintained in adult microbiomes [31, 32, 37]. Before adulthood the intestinal microbiome of children is still constantly remodeled and significantly different from adult individuals [37, 38]. Additionally, puberty is a major impact factor on the composition of the intestinal microbiome. The composition and abundance of certain taxa in adolescents was reported to significantly correlate with sex [39–41]. However, due to the relatively small size of our cohort, we have not split the respective age groups (age group 2 and 3) for sex, which is a limitation of our study considering the profound impact of hormones at that stage of live and the sex-bias of JIA.

The intestinal microbiota is a dynamic community that, in addition to age, can be affected by a variety of factors. In this study we have characterized a JIA cohort, which was relatively heterogeneous in regards to JIA subtypes, JIA-related therapy and antibiotic interventions. To assess the effect of these confounders, we investigated if the taxonomic and phenotypic data presents profound similarities of patients in that regard. However, we observed no significant difference for the factors therapy (Supp. Figure 10), subtype (Supp. Figure 11) as well as treatment with antibiotics in our cohort. We observed age-dependent similarities between healthy controls and JIA patients that corroborate age as a very relevant impact factor. These similarities are most prominent in the intestinal microbiota phenotype where we observed an overall trend of the intestinal microbiota to be characterized rather by lectin clusters in early age (age-group 1), a peak in immunoglobulin clusters in age-group 2 and a less complex selection of clusters in age-group 3. The taxonomic composition of healthy individuals was also hallmarked by the presence of different bacteria each age-group. Strikingly, we found that the general JIA signature (all patients vs. all controls) is biased towards the younger age groups and represents both, the healthy controls and the patients of higher age less well. By stratifying the JIA patients by age we identified age-specific taxonomic signatures (Fig. 2) and specific alterations in the composition of the JIA phenotype (Fig. 3), which were not observed without age stratification. Several taxonomic alterations found in our cohort are in line with results from previous studies e. g. an increase in Ruminococcaceae and *Veillonella,* whereas *Lachnospiracea pectinoschiza, Bacteroides sp., Akkermansia muciniphila, Monoglobus sp.* and *Phascolarctobacterium* were more enriched in healthy individuals [7, 34].

Moreover, we found distinct taxonomic signatures for each JIA age group (Fig. 2D) and only a small number of these taxa overlapped across the different age-stratified signatures or with the non-stratified JIA signature. We were able to identify additional taxa associated with JIA in an age-specific manner, such as the elevated abundance of *Bacteroides fragilis* and *Akkermansia sp.* in age group 1; *Sutterella sp., Parabacteroides merdae, Streptococcus sp., Romboutsia sp.* and *Enterococcus sp.* in age-group 2 and *Lachnospiraceae UCG-010 sp.* in age group 3. As we found several taxa to be uniquely associated with certain age groups, our data indicate that age stratification is crucial to understand the role of particular bacteria in the pathogenesis of JIA.

We also show age-specific alterations in the intestinal microbiota phenotype in JIA. We observed a phenotypic shift from increased agglutinin binding, dominantly by STL and PNA, towards an increase in immunoglobulin coating from age group 1 to age group 2. We speculate that this shift could be indicative of an increased immune recognition of the gut microbiota or a period of systemic effects of the local, chronic inflammation in JIA between the ages 6 to 11 years. Whether this is due to an elevated immune response in age group 2 to the increased abundance of *Sutterella sp., Parabacteroides merdae, Streptococcus sp., Romboutsia sp.* and *Enterococcus sp.*, could be addressed directly by sorting immunoglobulin-coated bacteria to gain a deeper understanding about their role in JIA pathogenesis as we and others have shown already in different contexts [42, 43].

## Conclusions

In conclusion, our study showed that age stratification of patients is crucial to improve the characterization of the intestinal microbiota in children with JIA. Further, we outline the extension of conventional sequencing by multi-parametric microbiota flow cytometry in clinical settings as a powerful tool to monitor phenotypic alterations in the host-microbiota crosstalk and the bacterial community, which will be essential to understand its role in JIA pathogenesis.

## Supplementary Information

**Supplementary Material: Figure S1.** Phenotyping of intestinal microbiota by multi-parameter microbiota flow cytometry and identification of disease-specific signatures. (A) Human intestinal bacteria from stool samples were stained with monoclonal antibodies specific for the human immunoglobulins IgA1, IgA2, IgM and IgG and with the lectins peanut agglutinin (PNA), wheat germ agglutinin (WGA), Solanum tuberosum lectin (STL) and Concanavalin A (ConA). Each staining panel also included the cell wall/membrane-permeable DNA dye Hoechst 33342. After data acquisition in a flow cytometer, the cells of each staining panel were clustered according to a previously defined self-organizing map (SOM) into 2025 clusters representing a set of phenotypic features and the abundance of cells that display those. The clusters for both panels are combined to compute the microbiota fingerprint out of 4050 clusters. The abundance of cells per cluster in the total of 4050 clusters represented the overall microbiota phenotype of a sample. (B) R-Pipeline to select cohort-specific features from the microbiota phenotype (B1) to obtain a microbiota biosignature (B2-B4). The clusters were filtered by (B2) Wilcoxon statistical evaluation and (B3) recursive feature elimination to select the significant and most robust clusters defining the specific microbiota phenotype signature represented for all samples by their beta-diversity (Bray-Curtis dissimilarity) projected by a Principal component Analysis (PCoA) (B4). In a PCoA the differences between samples correlate with their distance as more similar samples are closer to each other than very distinct samples (B4). The same analysis approach was used to identify taxonomic signatures for the cohort comparisons using a taxonomic count table as input. **Figure S2.** Taxonomic and phenotypic signature to identify JIA from pediatric controls before feature selection. PCoA of the beta-diversity by Bray-Curtis index of all samples of the JIA cohort (*n*=54 individuals, purple) and healthy controls (*n*= 38 individuals, grey) for the entire taxonomic signature(A) or phenotypic signature (B, 4050 clusters) of the intestinal microbiome for the comparison JIA vs. healthy pediatric controls. R2 and ANOVA test for each group. **Figure S3.** Detailed overview of selected taxonomic features to identify JIA from pediatric healthy controls.List of selected 28 taxonomic features ordered by generalized fold change. Features enriched in JIA (generalized fold change > 0) are shown in purple, features enriched in pediatric controls (generalized fold change < 0) are depicted in grey. The feature abundance is represented as box plot showing the 25th percentile, median and 75th percentile. **Figure S4.** Detailed overview of selected phenotypic clusters to identify JIA from healthy controls. (A) List of selected phenotypic clusters of both panels, ordered by generalized fold change and described for their marker composition by a heatmap (signal intensities normalized for each marker). (B) Representation of all selected phenotypic clusters for their location in 2D dot plot of all markers included in the panels (y-axis) against forward scatter (FSC, x-axis). All clusters enriched in JIA are depicted in purple, clusters enriched in controls are depicted in grey. **Figure S5**. Disease severity is no confounder of the taxonomic or phenotypic intestinal microbiota signatures in this JIA cohort. (A, B) Box plots for the distance of each patient Bray-Curtis beta-diversity index to the mean beta-diversity index of all healthy controls, evaluated for the disease severity (inactive/mild vs. high disease activity) intestinal microbiome signature in taxonomy (A) or microbiota phenotype (B). Grouped for disease severity: inactive/mild disease activity (IDa/MiDa, blue): cJADAS10 <4, moderate disease activity (MoDa, grey): cJADAS >4 and <13, high disease activity (HDa, red): cJADAS10>13. Box plot show median, upper hinge: 75th percentile, lower hinge 25th percentile of data. Wilcoxon test, unpaired. **Figure S6.** Detailed overview of selected taxonomic features to identify JIA from pediatric controls by age-wise comparison. (A, B, C) Box plots represent the abundance of all selected features for age group 1 (A, 9 taxa), age group 2 (B, 18 taxa) and age group 3 (C, 6 taxa) ordered by generalized fold change. Taxa enriched in JIA (generalized fold change > 0) are depicted in magenta (age group 1), blue (age group 2), dark green (age group 3) and taxa enriched in pediatric controls are depicted in pink (age group 1), light blue (age group 2), light green (age group 3). Box plot show median, upper hinge: 75th percentile, lower hinge 25th percentile of data. **Figure S7.** Representation of the selected phenotypic clusters for age-wise comparisons of JIA and pediatric controls. (A, B, C) Representation of all selected phenotypic clusters for their location in 2D dot plot of all markers included in the panels (y-axis) against forward scatter (FSC, x-axis). All clusters enriched in JIA are depicted in magenta (age group 1), blue (age group 2), dark green (age group 3) and clusters enriched in controls are depicted in pink (age group 1), light blue (age group 2), light green (age group 3). **Figure S8.** Multi-dimensional representation of the phenotypic clusters representing JIA by t-SNE. Combination of selected phenotypic clusters from all comparisons: all JIA vs. all pediatric controls plus age-matched comparisons (524 clusters). T-SNE shown for all markers normalized fluorescence intensities: blue = low intensity; red = high intensity. Clusters referring to immunoglobulin panel: rectangles. Clusters referring to lectin panel: circles. **Figure S9.** Microbiota signature of RA patients. (A, B) PCoA of the beta-diversity by Bray-Curtis index of all samples of the RA cohort (*n*=16 individuals, orange) and adult healthy controls (*n*= 18 individuals, beige) for the selected taxonomic signature (A, 20 features) or phenotypic signature (B, 72 clusters) of the intestinal microbiome for the comparison RA vs. adult controls. R2 and ANOVA test for each group. (C) Box plots represent the abundance of all 20 selected taxonomic features of the intestinal microbiome RA signature ordered by generalized fold change. Taxa enriched in RA (generalized fold change > 0) are depicted in orange and taxa enriched in adult controls are depicted beige. Box plot show median, upper hinge: 75th percentile, lower hinge 25th percentile of data. (D) Representation of all selected phenotypic clusters for their location in 2D dot plot of all markers included in the panels (y-axis) against forward scatter (FSC, x-axis). All clusters enriched in RA are depicted in orange, clusters enriched in controls are depicted in grey. **Figure S10.** Microbiota signatures of JIA therapeutics. (A, B) PCoA of the beta-diversity by Bray-Curtis index for all JIA patients (n=54) based on therapy for (A) 16S rRNA sequencing and (B) microbiota phenotyping. R2 and ANOVA test for therapy and ellipses indicate the 95 % confidence interval for each group. **Figure S11.** Microbiota signatures of JIA subtypes. (A, B) PCoA of the beta-diversity by Bray-Curtis index for all JIA patients (*n*=54) regarding subtype for (A) 16 SrRNA sequencing and (B) microbiota phenotyping. R2 and ANOVA test for subtype and ellipses indicate the 95 % confidence interval for each group.

## Data Availability

Fcs files & 16S rRNA gene sequencing data will be made accessible available after acceptance on Zenodo (10.5281/zenodo.13380537).

## Abbreviations

aHC: Adult healthy controls
JIA: Juvenile idiopathic arthritis
mMFC: Multi-parameter microbiota flow cytometry
PCoA: Principal coordinate analysis
pHC: Pediatric healthy controls
RA: Rheumatoid arthritis
SOM: Self-organizing map
AUROC: Area under the receiver operator curve
RFE: Recursive feature elimination
