# Supplementary material for: Age-stratification reveals age-specific intestinal microbiota signatures in juvenile idiopathic arthritis
Source: Mol Cell Pediatr. 2024 Dec 10;11:12. doi: 10.1186/s40348-024-00186-6 (PMC11628465; doi:10.1186/s40348-024-00186-6)

Figure 1

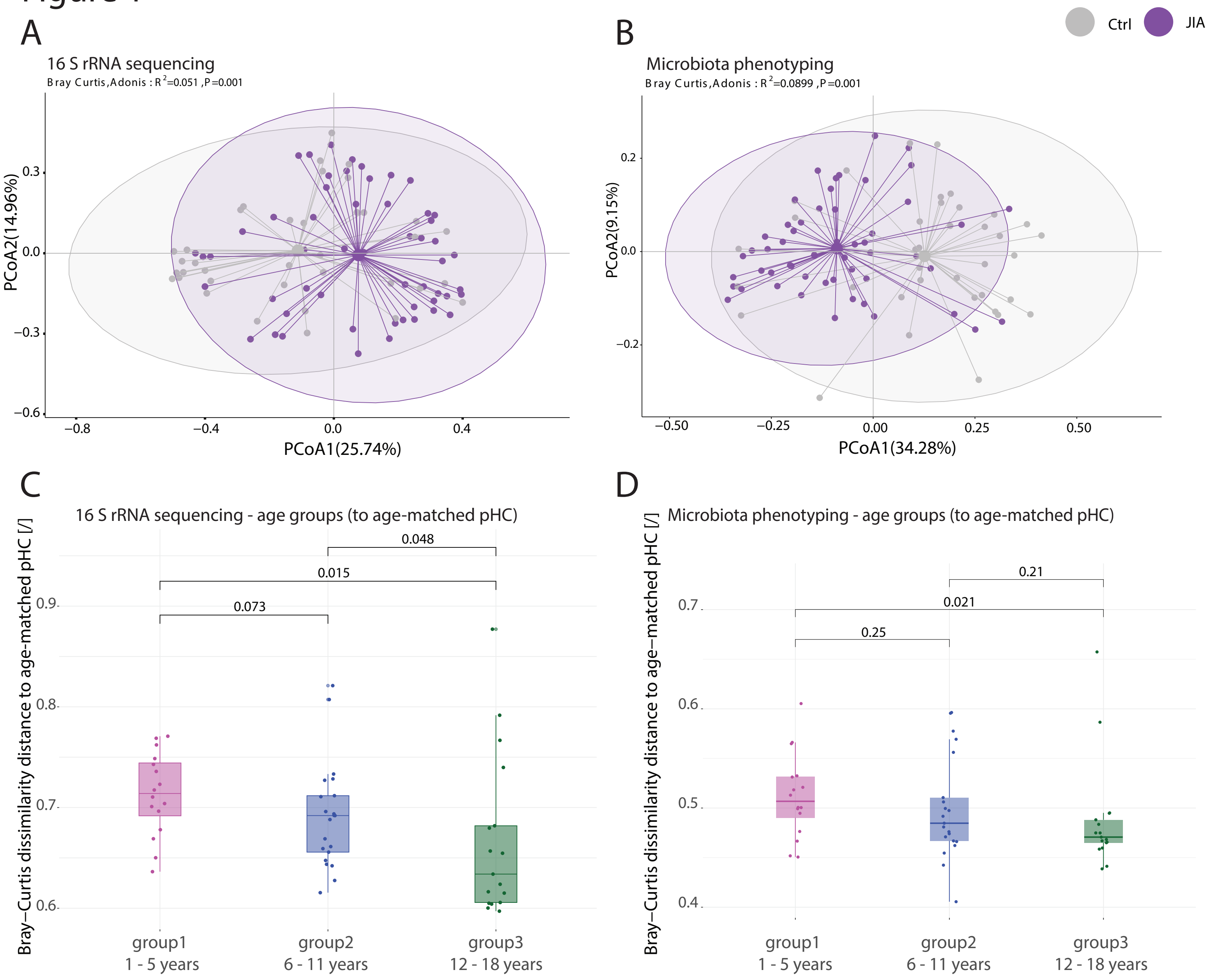
A
16 S rRNA sequencing
Bray Curtis,Adonis : R^2=0.051 ,P=0.001
PCoA2(14.96%)
PCoA1(25.74%)
B
Microbiota phenotyping
Bray Curtis,Adonis : R^2=0.0899 ,P=0.001
PCoA2(9.15%)
PCoA1(34.28%)
Ctrl
JIA
C
16 S rRNA sequencing - age groups (to age-matched pHC)
Bray–Curtis dissimilarity distance to age-matched pHC [/]
0.048
0.015
0.073
group1 1 - 5 years
group2 6 - 11 years
group3 12 - 18 years
D
Microbiota phenotyping - age groups (to age-matched pHC)
Bray–Curtis dissimilarity distance to age-matched pHC [/]
0.21
0.021
0.25
group1 1 - 5 years
group2 6 - 11 years
group3 12 - 18 years

Figure 2

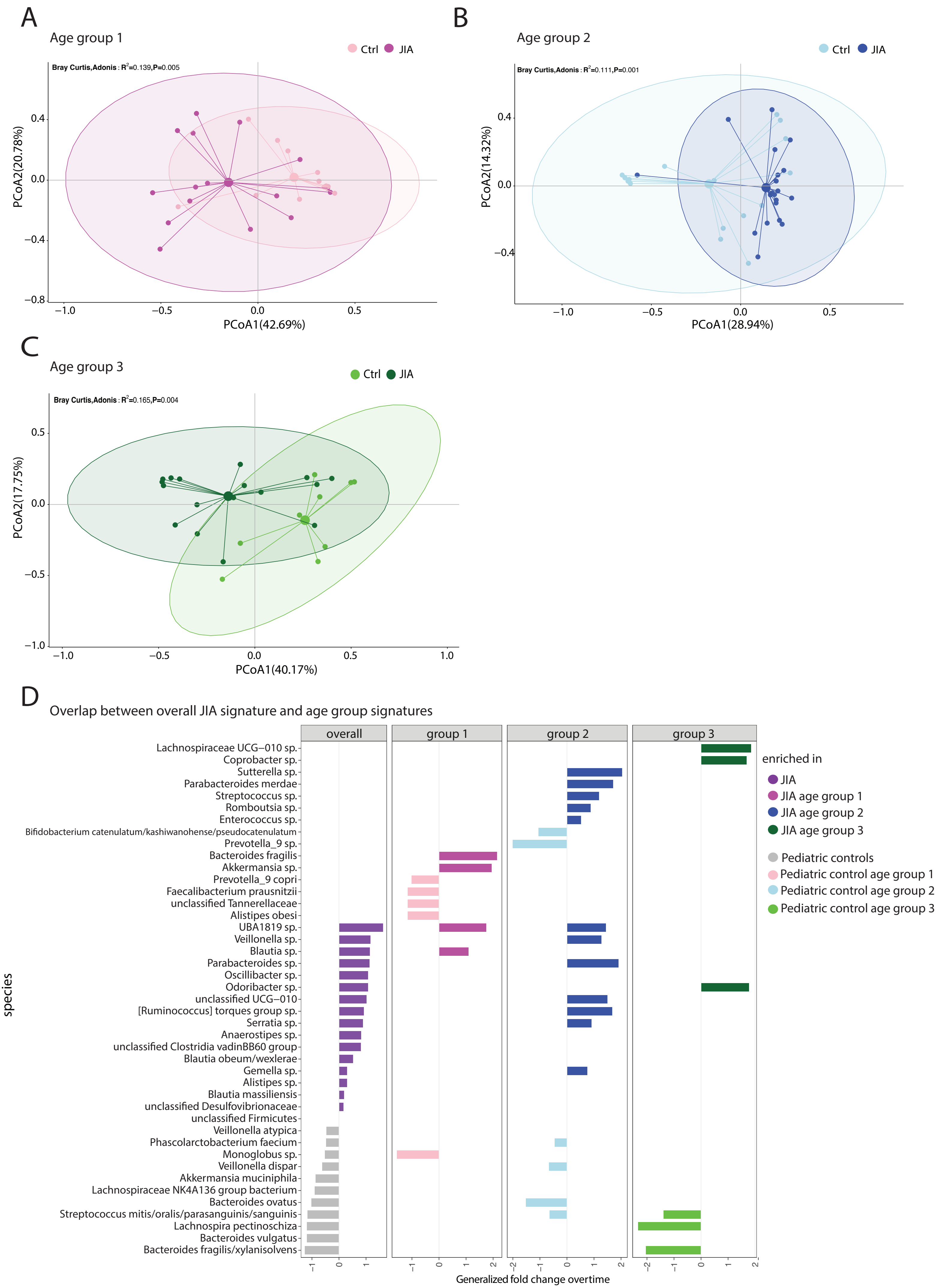

A
Age group 1
Ctrl
JIA
Bray Curtis,Adonis : R^2=0.139,P=0.005
PCoA2(20.78%)
PCoA1(42.69%)
0.4
0.0
−0.4
−0.8
−1.0
−0.5
0.0
0.5
B
Age group 2
Ctrl
JIA
Bray Curtis,Adonis : R^2=0.111,P=0.001
PCoA2(14.32%)
PCoA1(28.94%)
0.4
0.0
−0.4
−1.0
−0.5
0.0
0.5
C
Age group 3
Ctrl
JIA
Bray Curtis,Adonis : R^2=0.165,P=0.004
PCoA2(17.75%)
PCoA1(40.17%)
0.5
0.0
−0.5
−1.0
−1.0
−0.5
0.0
0.5
1.0
D
Overlap between overall JIA signature and age group signatures
overall
group 1
group 2
group 3
species
Lachnospiraceae UCG−010 sp.
Coprobacter sp.
Sutterella sp.
Parabacteroides merdae
Streptococcus sp.
Romboutsia sp.
Enterococcus sp.
Bifidobacterium catenulatum/kashiwanohense/pseudocatenulatum
Prevotella_9 sp.
Bacteroides fragilis
Akkermansia sp.
Prevotella_9 copri
Faecalibacterium prausnitzii
unclassified Tannerellaceae
Alistipes obesi
UBA1819 sp.
Veillonella sp.
Blautia sp.
Parabacteroides sp.
Oscillibacter sp.
Odoribacter sp.
unclassified UCG−010
[Ruminococcus] torques group sp.
Serratia sp.
Anaerostipes sp.
unclassified Clostridia vadinBB60 group
Blautia obeum/wexlerae
Gemella sp.
Alistipes sp.
Blautia massiliensis
unclassified Desulfovibrionaceae
unclassified Firmicutes
Veillonella atypica
Phascolarctobacterium faecium
Monoglobus sp.
Veillonella dispar
Akkermansia muciniphila
Lachnospiraceae NK4A136 group bacterium
Bacteroides ovatus
Streptococcus mitis/oralis/parasanguinis/sanguinis
Lachnospira pectinoschiza
Bacteroides vulgatus
Bacteroides fragilis/xylanisolvens
Generalized fold change overtime
enriched in
JIA
JIA age group 1
JIA age group 2
JIA age group 3
Pediatric controls
Pediatric control age group 1
Pediatric control age group 2
Pediatric control age group 3

Figure 3

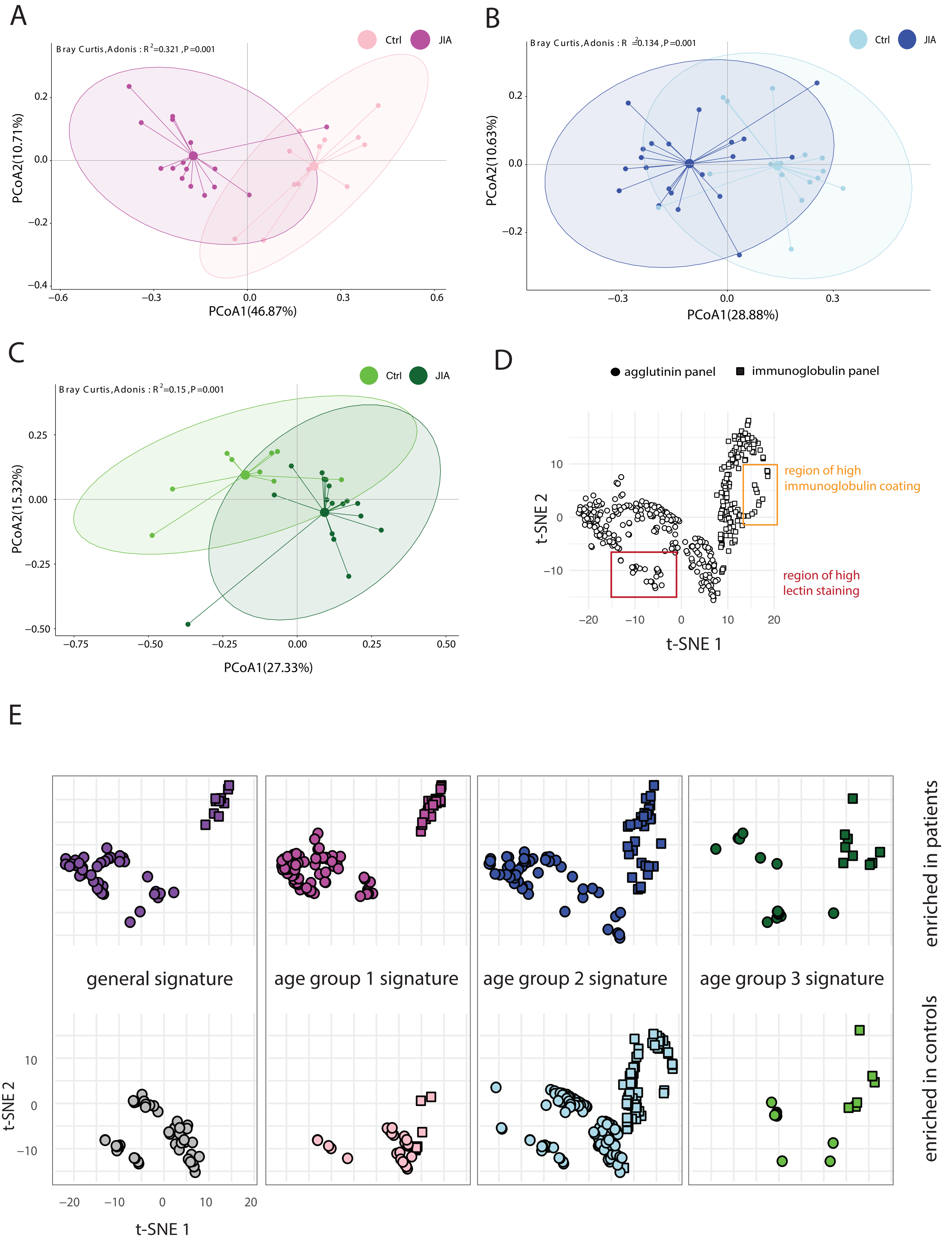
A
Bray Curtis, Adonis : R²=0.321 , P=0.001
Ctrl
JIA
PCoA2(10.71%)
PCoA1(46.87%)
B
Bray Curtis, Adonis : R²=0.134 , P=0.001
Ctrl
JIA
PCoA2(10.63%)
PCoA1(28.88%)
C
Bray Curtis, Adonis : R²=0.15 , P=0.001
Ctrl
JIA
PCoA2(15.32%)
PCoA1(27.33%)
D
agglutinin panel
immunoglobulin panel
region of high immunoglobulin coating
region of high lectin staining
t-SNE 2
t-SNE 1
E
general signature
age group 1 signature
age group 2 signature
age group 3 signature
enriched in patients
enriched in controls
t-SNE 2
t-SNE 1

Figure 4

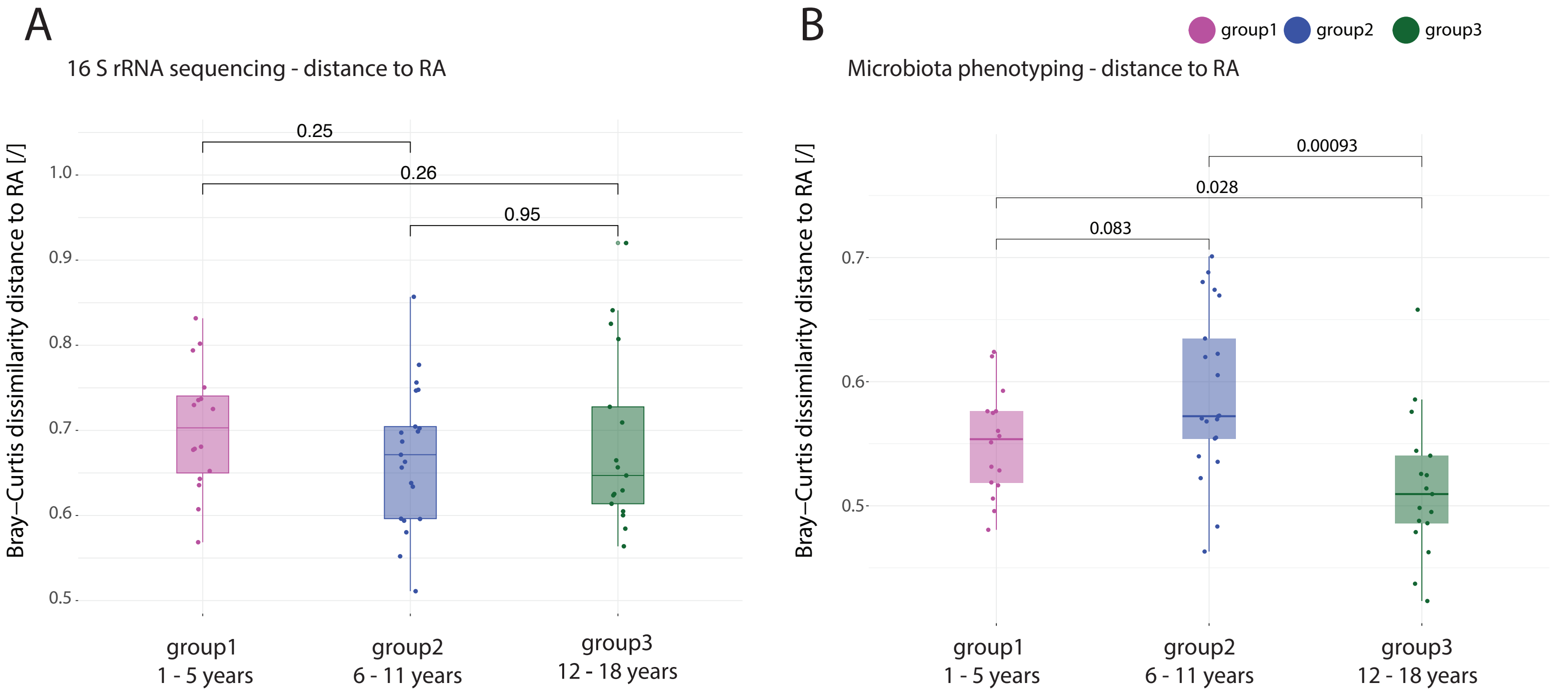

A
16 S rRNA sequencing - distance to RA
Bray–Curtis dissimilarity distance to RA [/]
0.25
0.26
0.95
1.0
0.9
0.8
0.7
0.6
0.5
group1
1 - 5 years
group2
6 - 11 years
group3
12 - 18 years
B
group1
group2
group3
Microbiota phenotyping - distance to RA
Bray–Curtis dissimilarity distance to RA [/]
0.00093
0.028
0.083
0.7
0.6
0.5
group1
1 - 5 years
group2
6 - 11 years
group3
12 - 18 years

Supplementary Figure 1

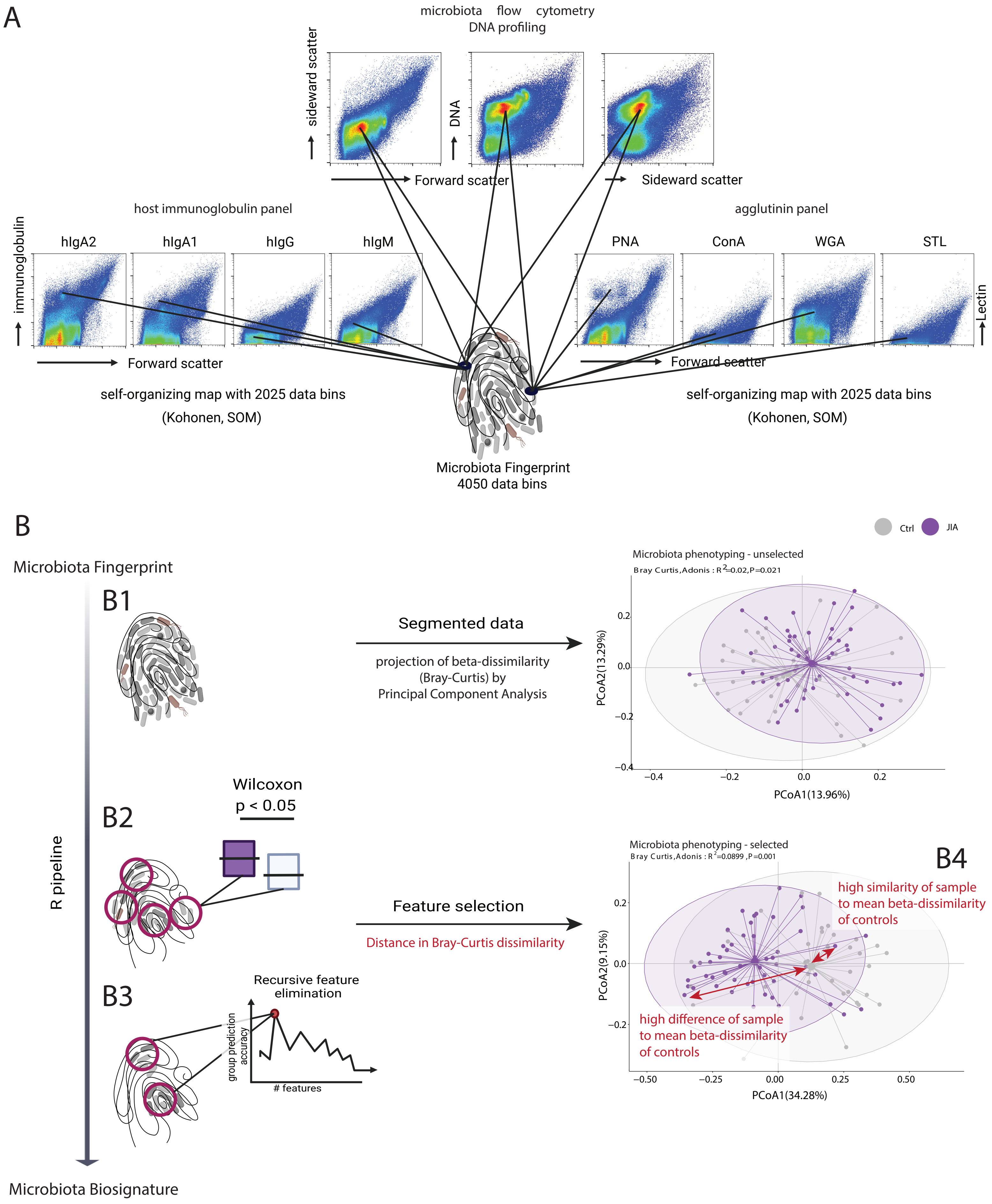

Supplementary Figure 2

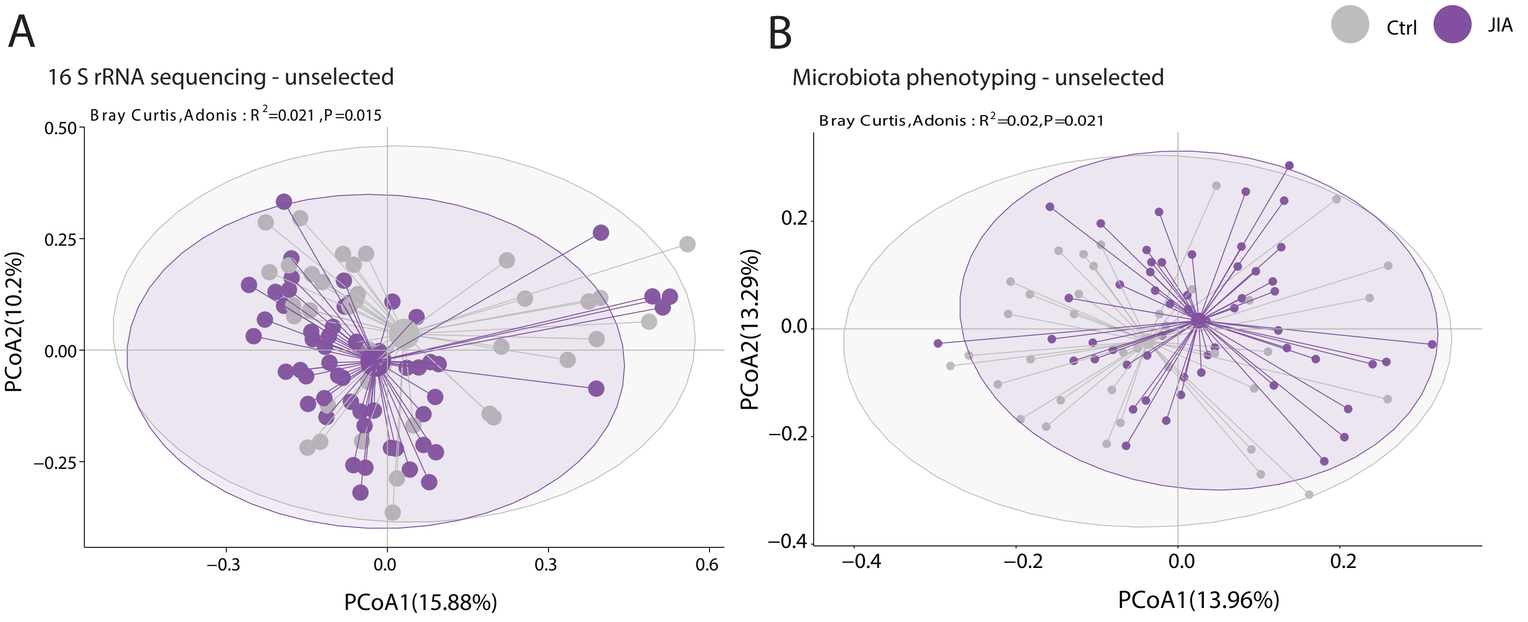

Supplementary Figure 3

16S rRNA sequencing - selected species

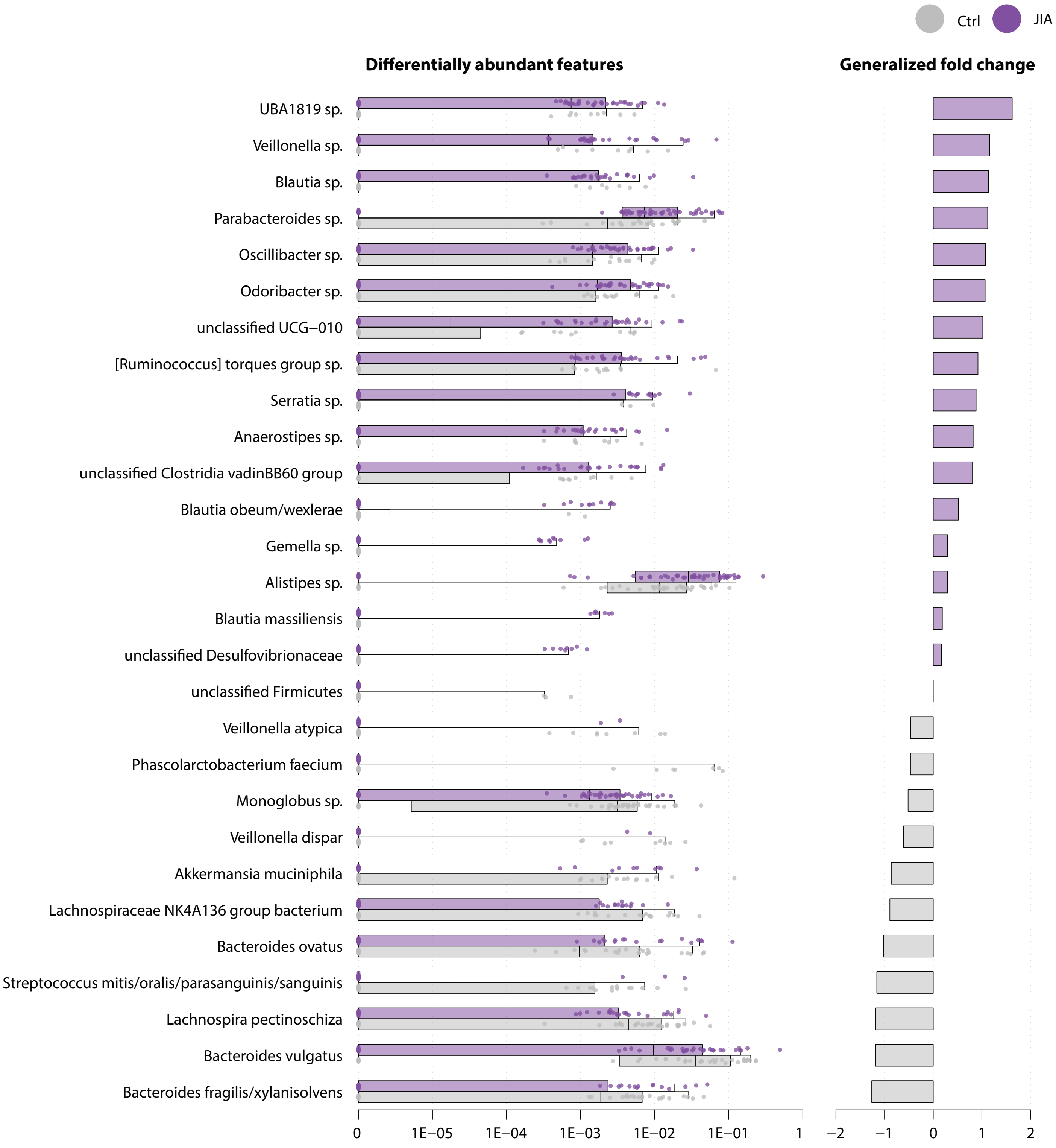

Supplementary Figure 4

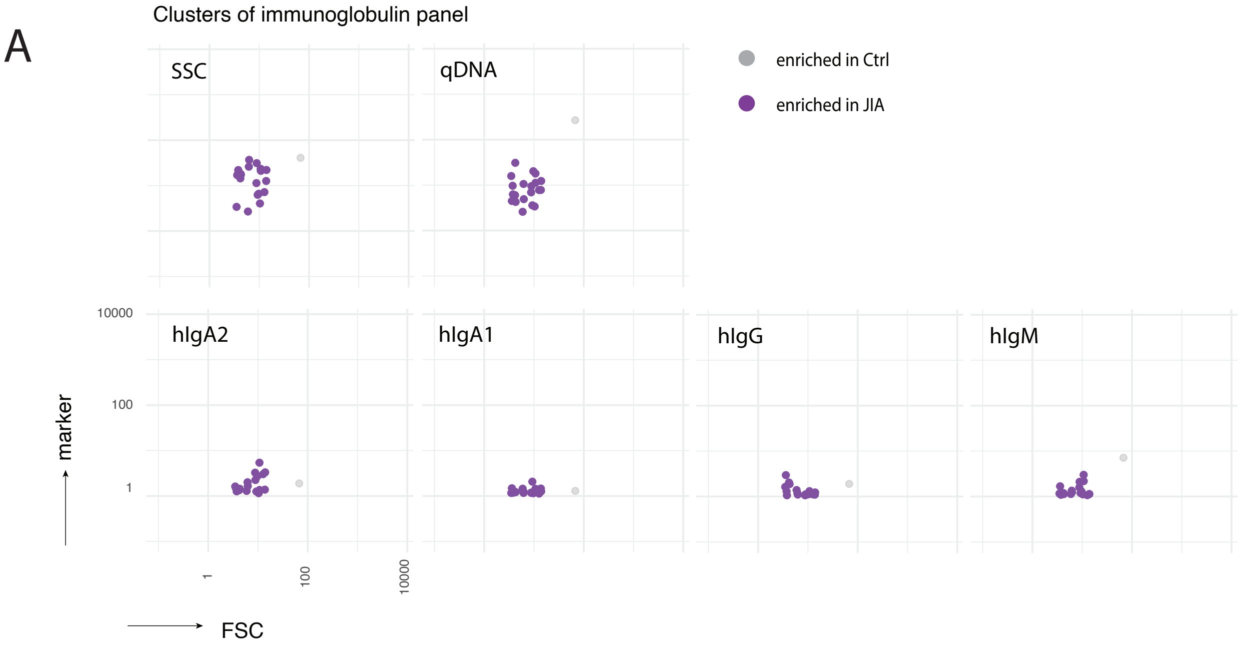

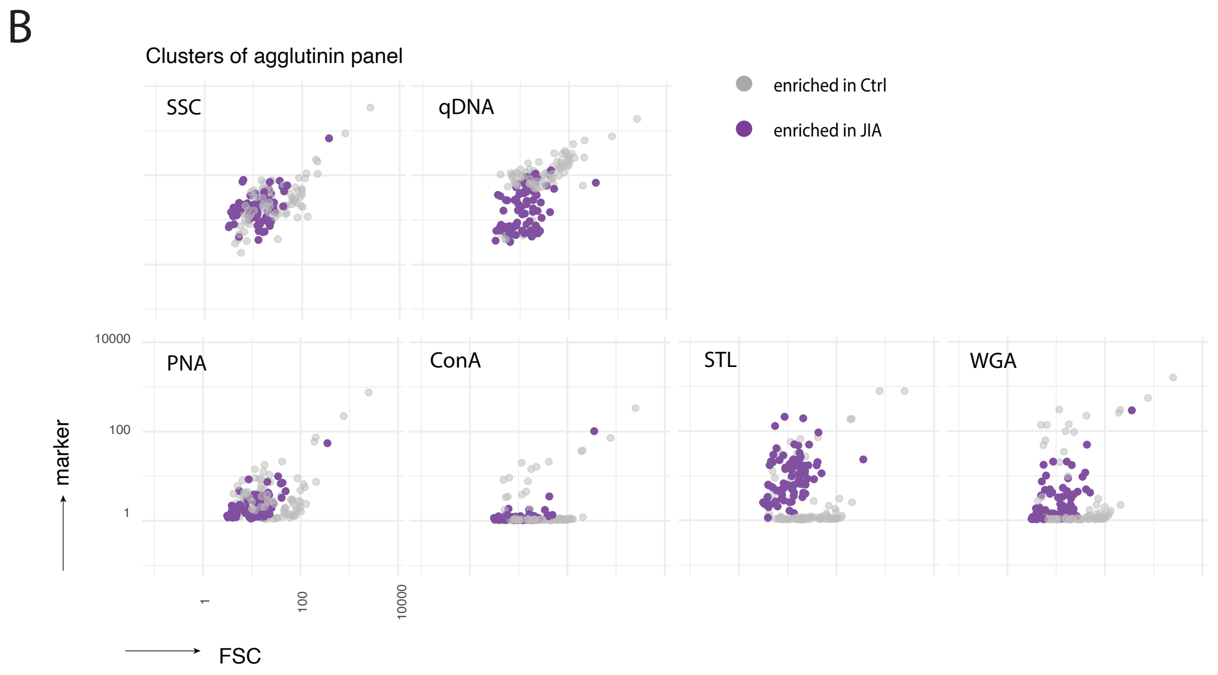

Supplementary Figure 5

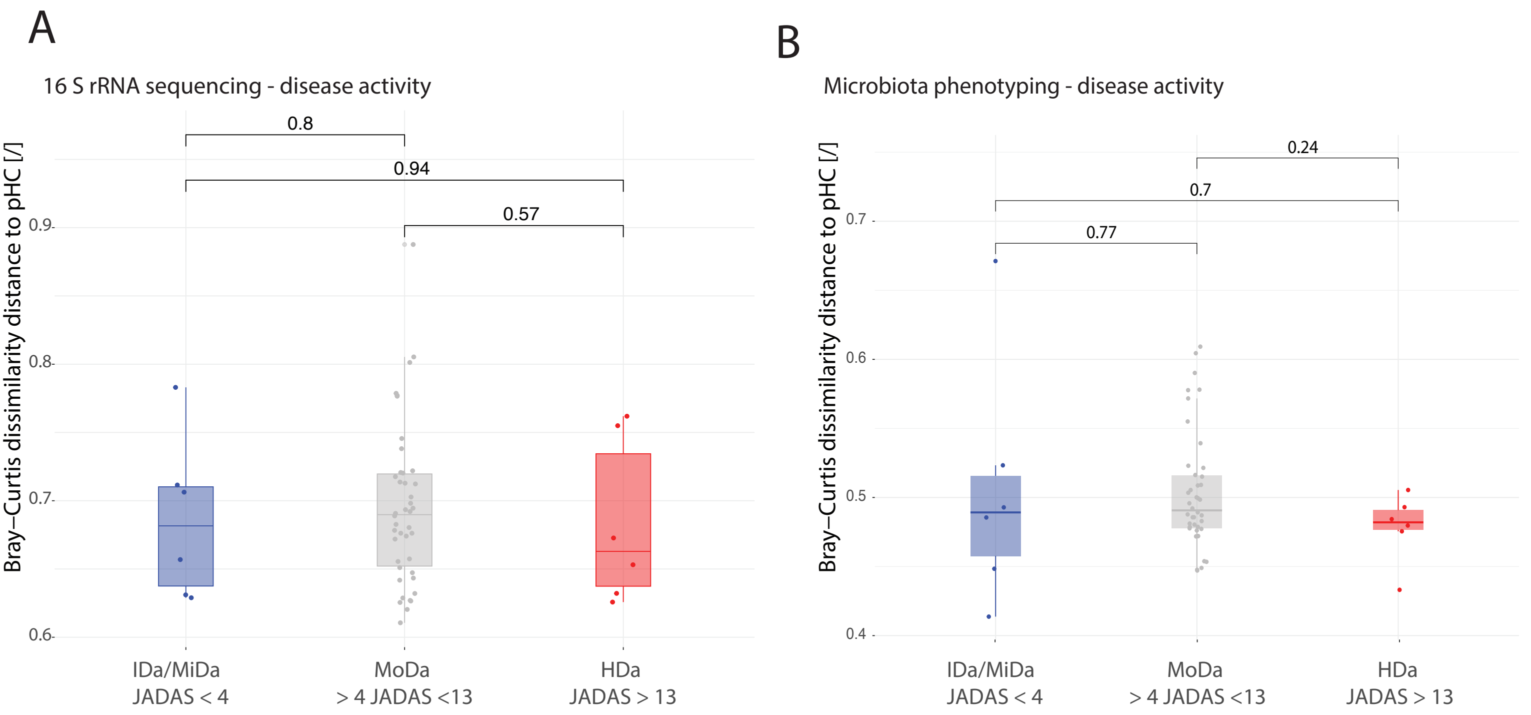

Supplementary Figure 6

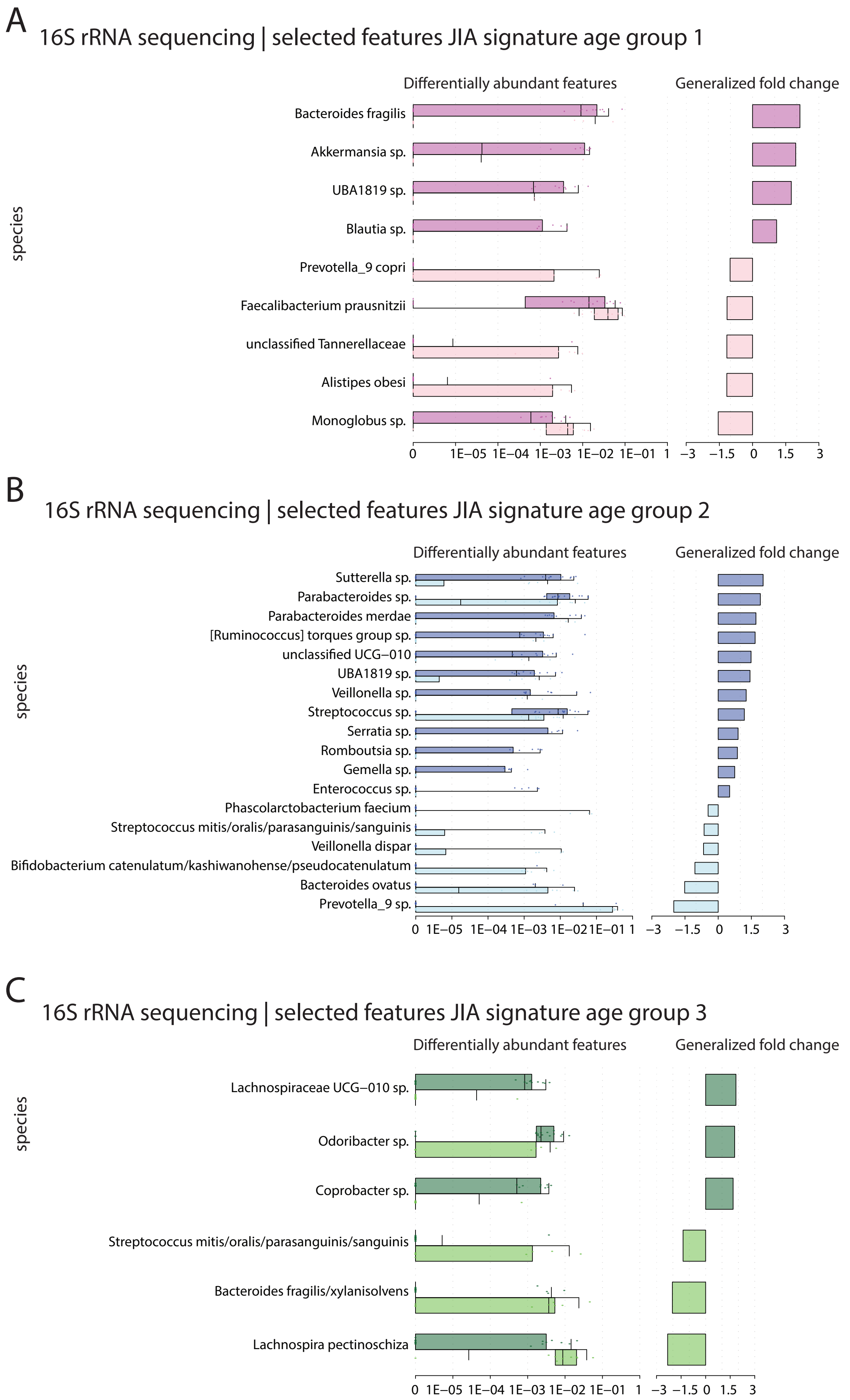

Supplementary Figure 7

A

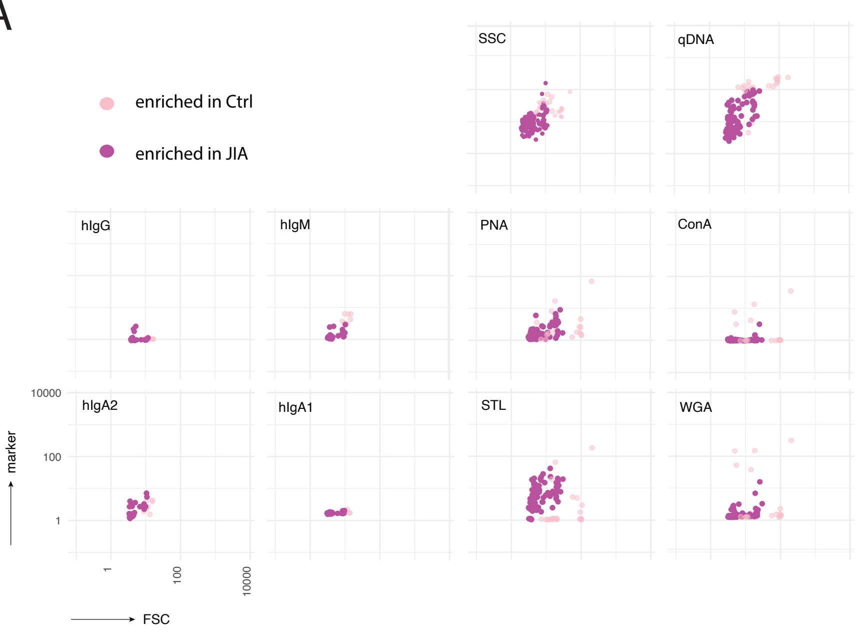

B

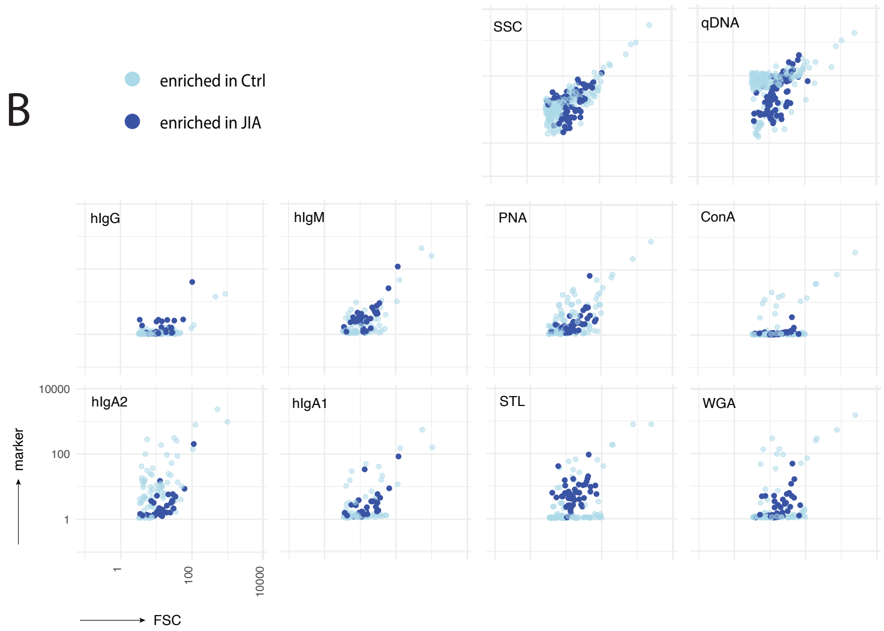

C

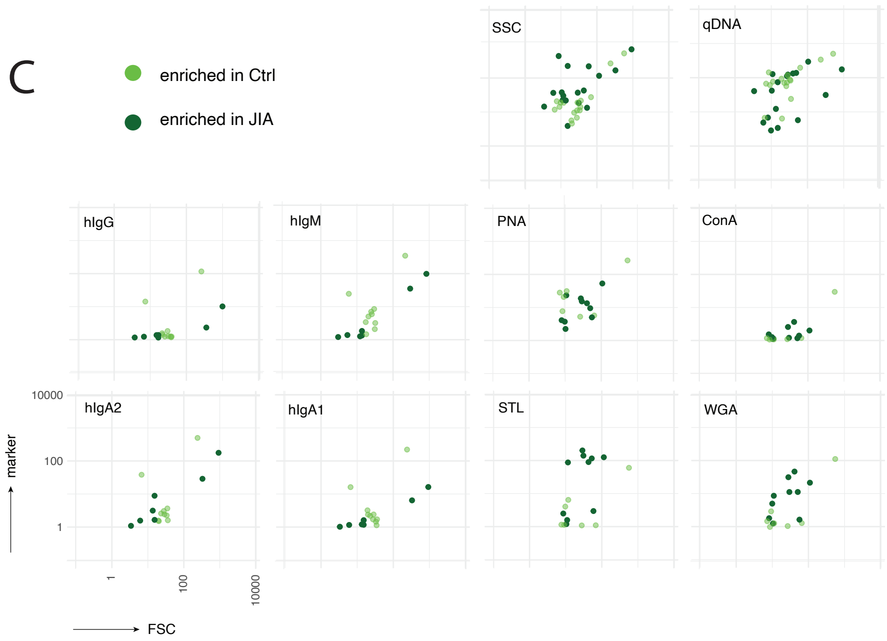

Supplementary Figure 8

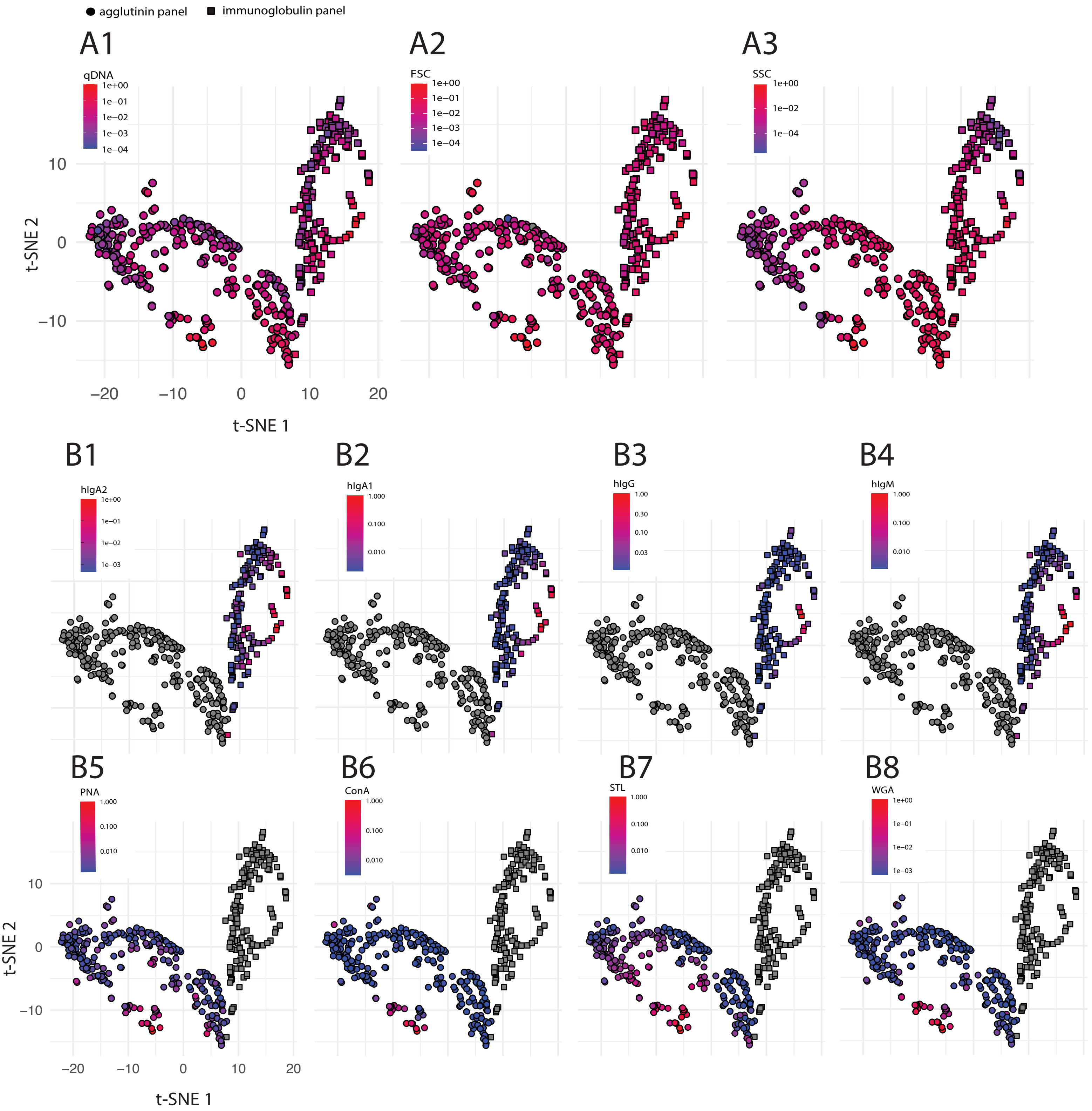

Supplementary Figure 9

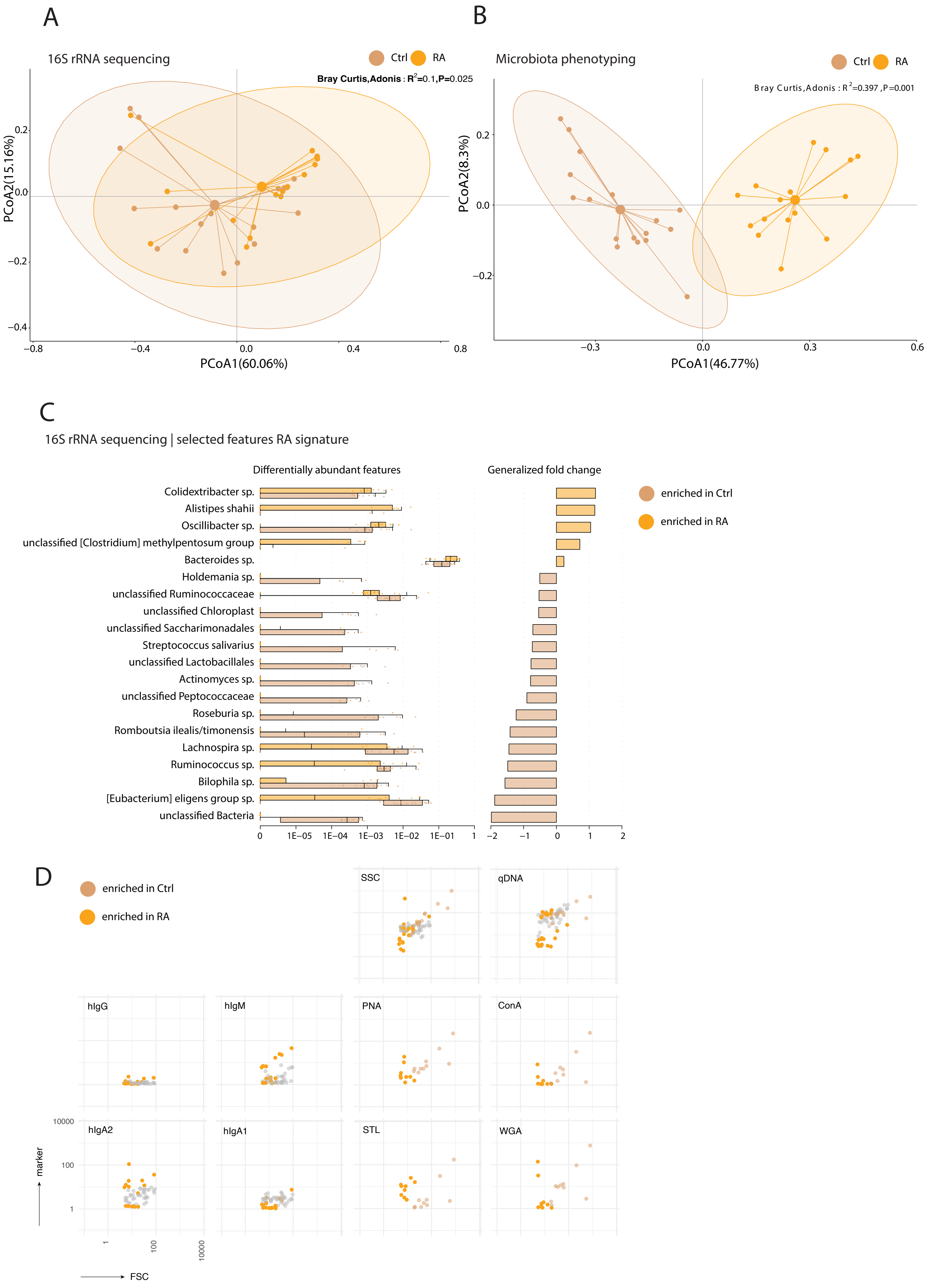

Supplementary Figure 10

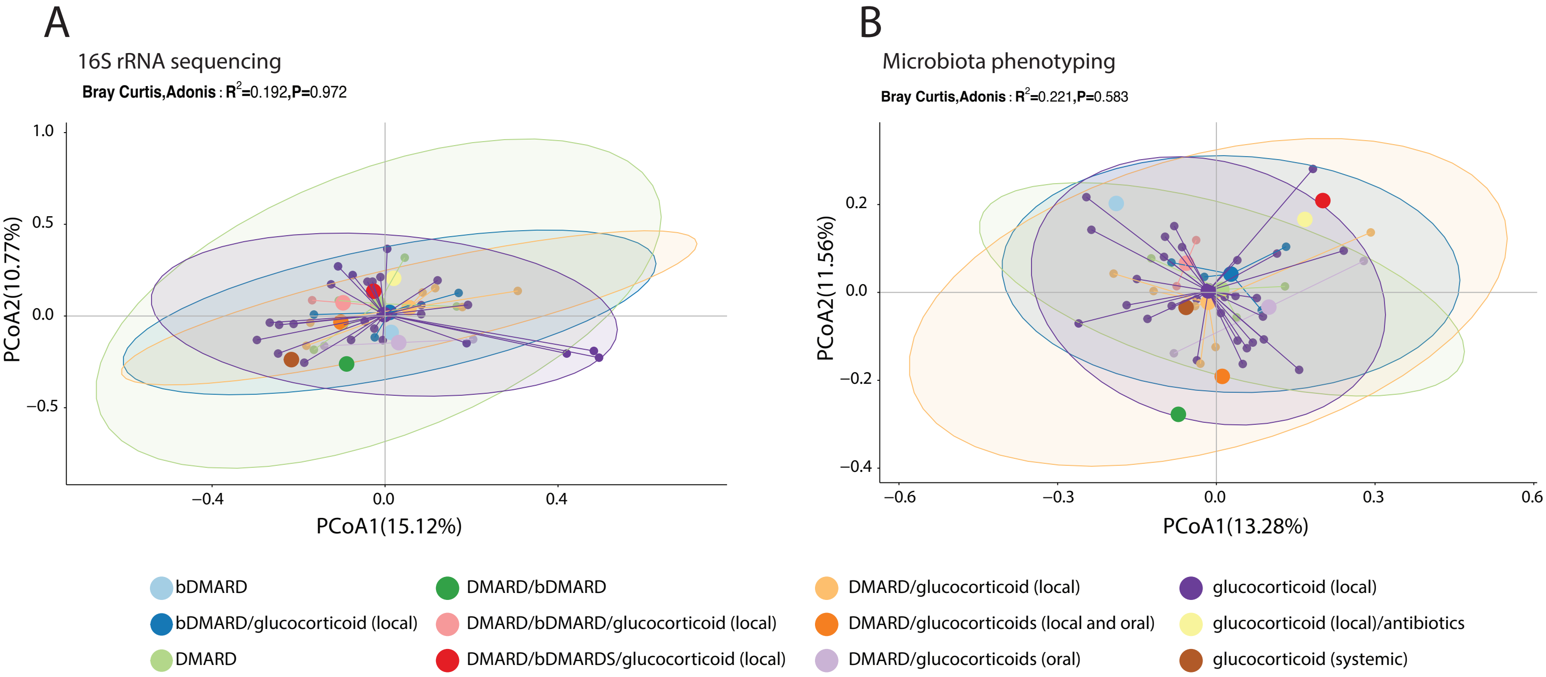

Supplementary Figure 11

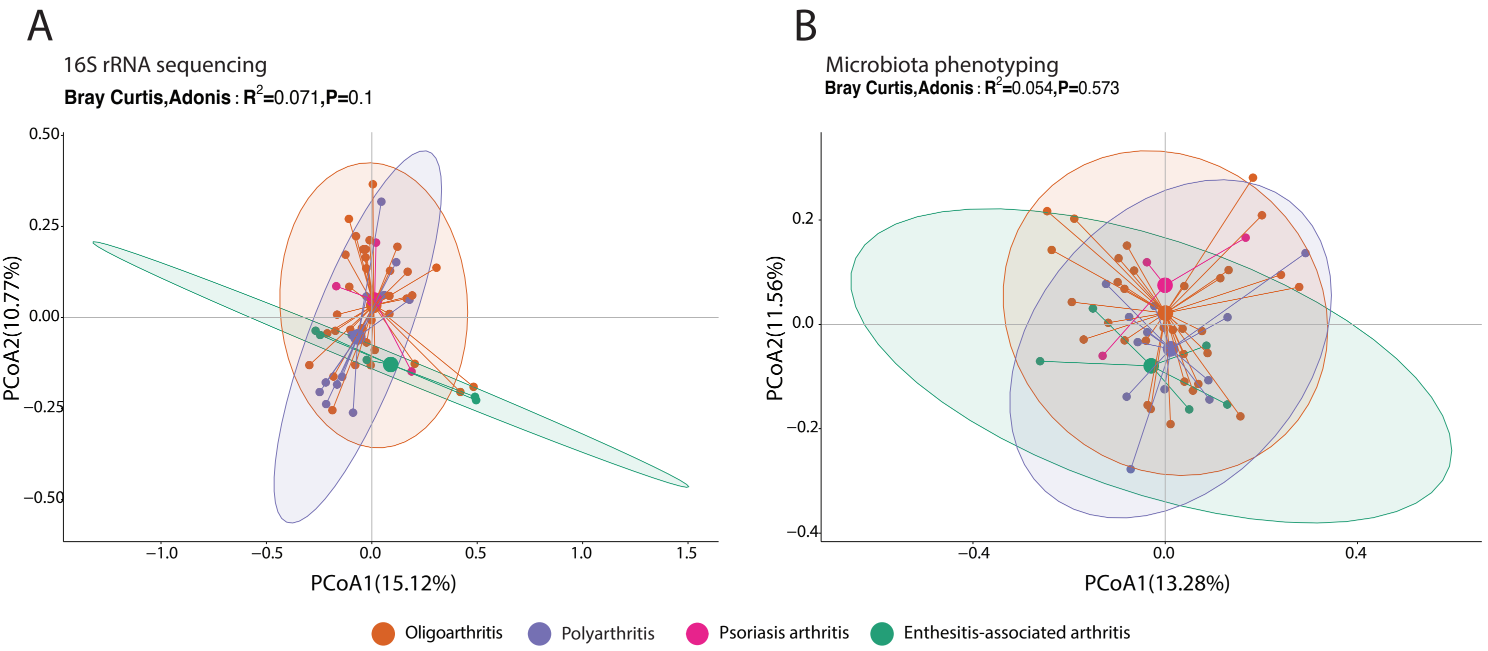

Supplement: Supplementary file 1 — Supplementary Material: Figure S1. Phenotyping of intestinal microbiota by multi-parameter microbiota flow cytometry and identification of disease-specific signatures. (A) Human intestinal bacteria from stool samples were stained with monoclonal antibodies specific for the human immunoglobulins IgA1, IgA2, IgM and IgG and with the lectins peanut agglutinin (PNA), wheat germ agglutinin (WGA), Solanum tuberosum lectin (STL) and Concanavalin A (ConA). Each staining panel also included the cell wall/membrane-permeable DNA dye Hoechst 33342. After data acquisition in a flow cytometer, the cells of each staining panel were clustered according to a previously defined self-organizing map (SOM) into 2025 clusters representing a set of phenotypic features and the abundance of cells that display those. The clusters for both panels are combined to compute the microbiota fingerprint out of 4050 clusters. The abundance of cells per cluster in the total of 4050 clusters represented the overall microbiota phenotype of a sample. (B) R-Pipeline to select cohort-specific features from the microbiota phenotype (B1) to obtain a microbiota biosignature (B2-B4). The clusters were filtered by (B2) Wilcoxon statistical evaluation and (B3) recursive feature elimination to select the significant and most robust clusters defining the specific microbiota phenotype signature represented for all samples by their beta-diversity (Bray-Curtis dissimilarity) projected by a Principal component Analysis (PCoA) (B4). In a PCoA the differences between samples correlate with their distance as more similar samples are closer to each other than very distinct samples (B4). The same analysis approach was used to identify taxonomic signatures for the cohort comparisons using a taxonomic count table as input. Figure S2. Taxonomic and phenotypic signature to identify JIA from pediatric controls before feature selection. PCoA of the beta-diversity by Bray-Curtis index of all samples of the JIA cohort (n=54 indivi [file 40348_2024_186_MOESM1_ESM.pdf]
